# Supplementary material for: BEAR reveals that increased fidelity variants can successfully reduce the mismatch tolerance of adenine but not cytosine base editors
Source: Nat Commun. 2021 Nov 3;12:6353. doi: 10.1038/s41467-021-26461-y (PMC8566456; doi:10.1038/s41467-021-26461-y)
Supplement: Supplementary file 1 — Supplementary Information [file 41467_2021_26461_MOESM1_ESM.pdf]

Supplementary Information

for

BEAR reveals that increased fidelity variants can successfully reduce the mismatch-tolerance of adenine but not cytosine base editors

András Tálas*, Dorottya A. Simon, Péter I. Kulcsár, Éva Varga, Sarah L. Krausz, Ervin Welker*

* Corresponding author. E-mail: talas.andras@ttk.hu, welker.ervin@ttk.hu

Contents:

Supplementary Figure 1 – Canonical and non-canonical splice donor sites examined in BEAR...................... 2

Supplementary Figure 2 – Splice site variants for identifying candidate BEAR sequences in N2a cells.......... 2

Supplementary Figure 3 – Versatility of BEAR allows its compatibility with different sequences................. 3

Supplementary Figure 4 – Enrichment of base edited cells with nickase ABE 4

Supplementary Figure 5 – Scatter plots of the editing efficiencies of ABE and CBE variants 5

Supplementary Figure 6 – Activity of base editor variants with 50 mismatching sgRNAs.............................. 5

Supplementary Figure 7 – Off-target activities of different ABE variants on three additional targets with 1 matched and 31 mismatched sgRNAs 6

Supplementary Figure 8 – The activities of ABE8e variants are more similar to CBE than to ABE 8

Supplementary Figure 9 – Bystander editing of ABE8e 9

Supplementary Figure 10 – The on-target activity of dABE barely correlates with evo- or HeF-ABE 9

Supplementary Figure 11 – Flow cytometry gating examples 11

Supplementary Methods – Detailed plasmid construction 13

Supplementary Table 1 – Comparison of seven fluorescence-based markers of base editing......................... 17

Supplementary Table 2 – List of oligonucleotides 18

Supplementary Table 3 – List of NGS primers and NGS indexing 31

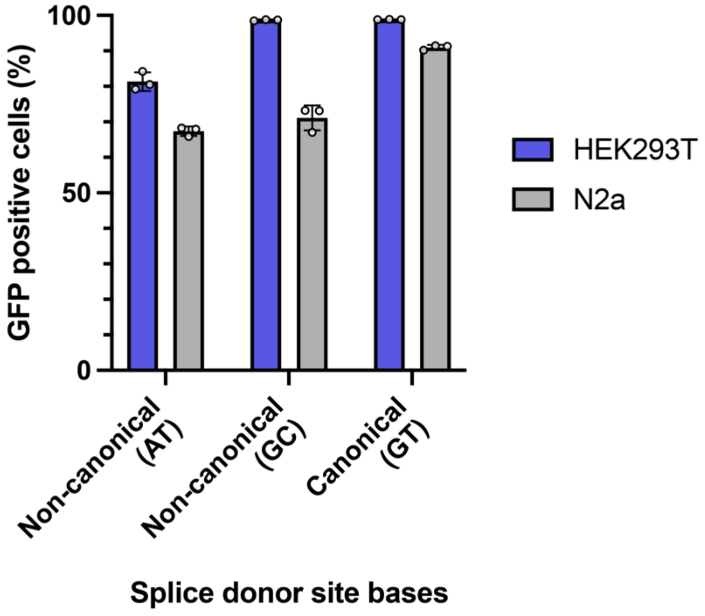

Supplementary Figure 1 – Canonical and non-canonical splice donor sites examined in BEAR

Canonical (GT) or non-canonical (AT or GC) splice donor sites were inserted into the split GFP coding plasmid (**Fig. 1**) and were transformed either into HEK293T (blue) or N2a (grey) cells. Columns represent means +/- SD of three parallel transfections (grey circles).

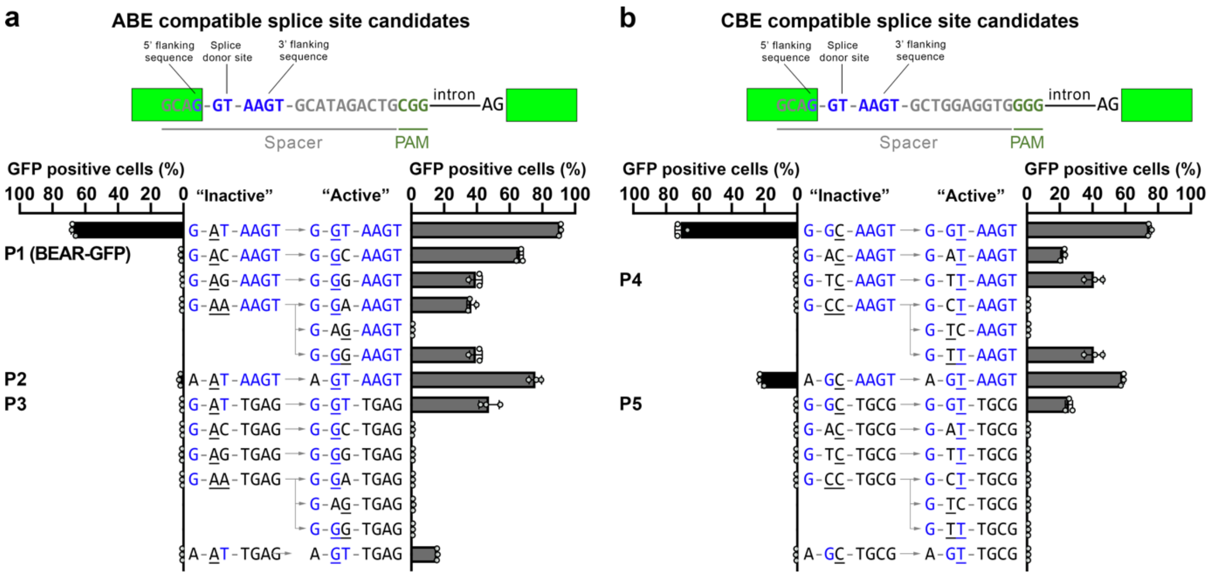

Supplementary Figure 2 – Splice site variants for identifying candidate BEAR sequences in N2a cells

Flow cytometry measurements of GFP positive N2a cells, transfected with plasmids harbouring systematically altered splice sites (expected "inactive" sequences), which can be converted by ABE (**a**) or CBE (**b**) to sequences expected to be functional, "active" splice sites. The sequences between the column charts represent the intended "inactive" or "active" splice site sequence pairs. Letters highlighted in blue indicate the bases that correspond to the canonical splice donor site and the flanking sequences: 5' - G GT AAGT - 3' (upper panels); the altered bases to be edited in the splice sites are

underlined. Five sequence pairs (P1-P5) with minimal fluorescence for the inactive and maximal fluorescence for the active splice donor site were selected for further analyses as detailed in **Fig. 2c-e.** Columns represent means, +/- SD of three parallel transfections (grey circles).

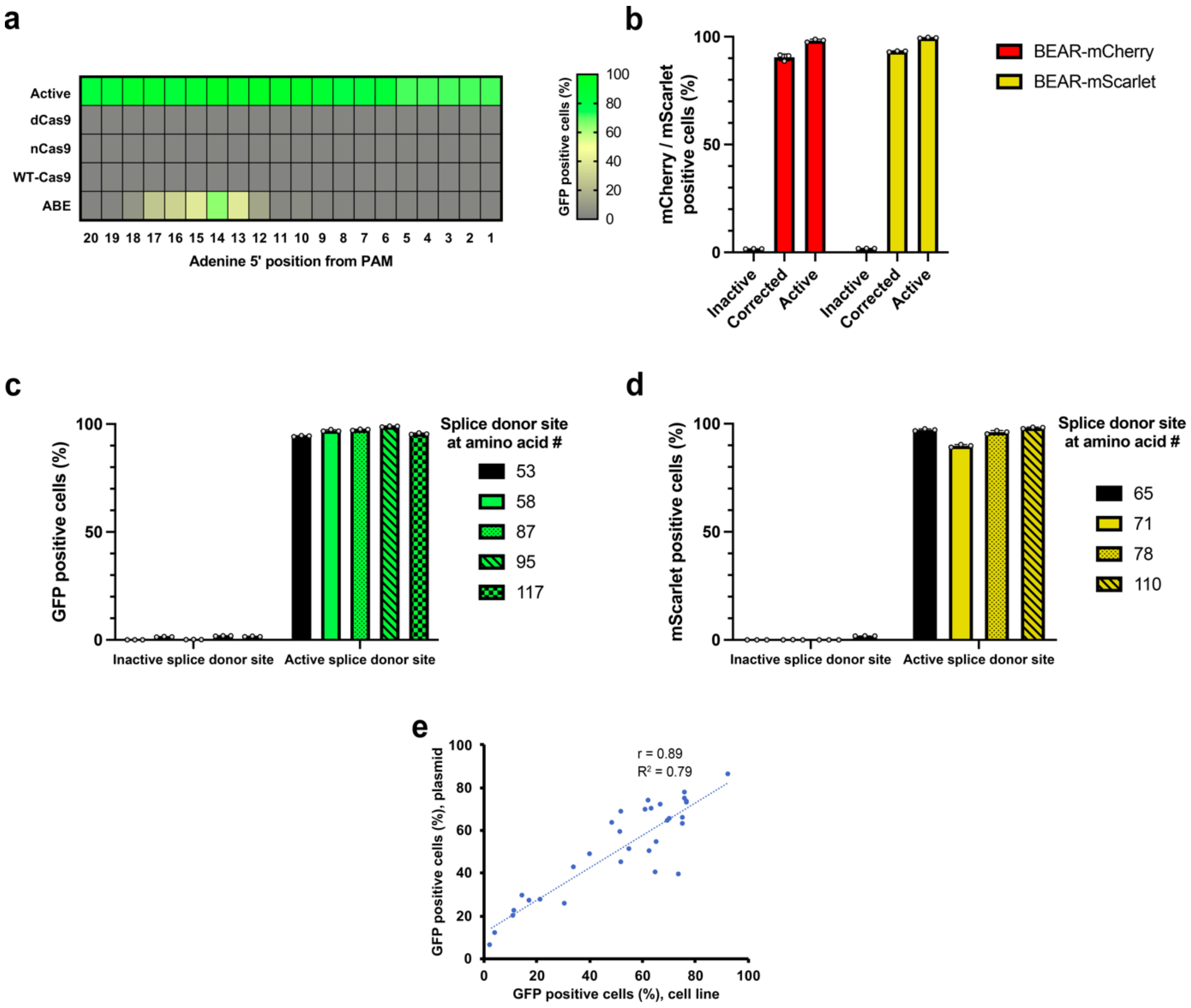

Supplementary Figure 3 – Versatility of BEAR allows its compatibility with different sequences

(**a**) Heatmap showing the percentage of GFP positive HEK293T cells transfected with various constructs, in which the PAM motif is shifted in relation to the base to be edited so that the edited base can occupy any position along the spacer sequence, thus, the editing window of ABE can be monitored. Each spacer contains only one adenine base and that adenine is the target. As a positive control for each edited position, a plasmid with an active splice site was constructed. As negative controls, constructs with inactive splice donor sites were co-transfected with the corresponding sgRNAs and either dead, nickase or nuclease (WT) SpCas9. Data is derived from three parallel transfections.

(**b**) HEK293T cells were transfected with plasmids harbouring an inactive splice site along with ABE (corrected) or along with nSpCas9 as negative control (inactive). As positive controls, plasmids with active splice sites were also transfected to monitor the maximum theoretical extent of base editing. BEAR-mCherry (red) and BEAR-mScarlet (yellow) contain the exact same intron and splice site that were used with BEAR-GFP. Columns represent means +/- SD of three parallel transfections (grey circles).

HEK293T cells are transfected with plasmids harbouring a GFP **(c)** and mScarlet **(d)** sequence interrupted with the intron at different amino acid positions within their sequence as indicated in the figure. All constructs expressed high levels of GFP and mScarlet, with all intron positions. Columns represent means +/- SD of three parallel transfections (grey circles).

(**e**) Scatter plot of GFP positive cells measured either when the BEAR-GFP plasmid or the BEAR-GFP cell line was edited with one matching and 32 mismatching sgRNAs (Pearson's r=0.89).

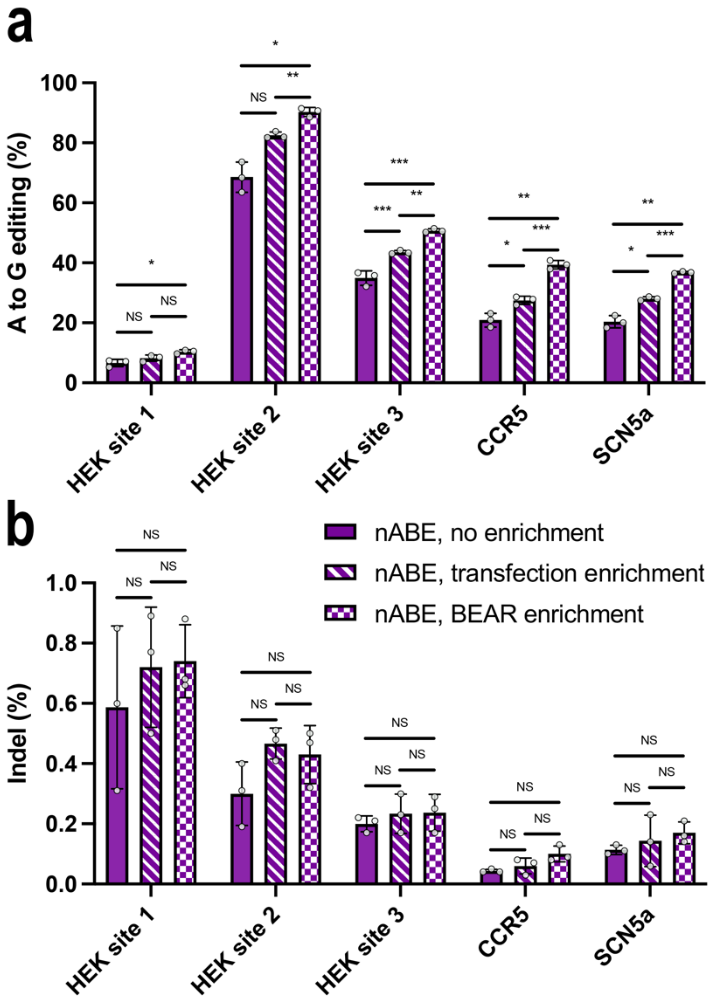

Supplementary Figure 4 – Enrichment of base edited cells with nickase ABE

The BEAR-GFP-2in1 plasmid and endogenous genomic targets were co-edited by nABE and analysed by NGS. Edited cells were sorted to 3 fractions: all cells (no enrichment, plain purple), BFP positive cells (transfection enrichment, striped purple), and cells with GFP positivity representing base editing enriched cells (BEAR enrichment, chequered purple). Base editing (**a**) and indel formation (**b**) were quantified from the same samples. Columns represent means +/- SD of three parallel transfections (grey circles). Differences between samples were tested using one-way ANOVA. NS:p>0.05, *:p<0.05, **:p<0.01, ***:p<0.001. For source data and exact p-values see Supplementary Data.

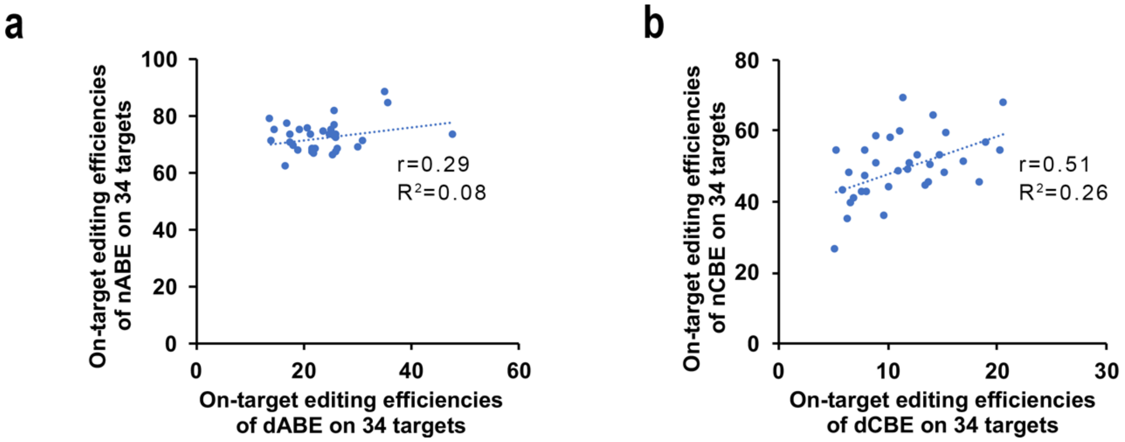

Supplementary Figure 5 – Scatter plots of the editing efficiencies of ABE and CBE variants

(**a**) Scatter plot for 34 on-target editing results (see **Figure 5**) when the nuclease inactive (dABE) and nickase ABE (nABE) are compared (Pearson's r=0.29).

(**b**) Scatter plot for 34 on-target editing results (see **Figure 5**) when the nuclease inactive (dCBE) and nickase CBE (nCBE) are compared (Pearson's r=0.51).

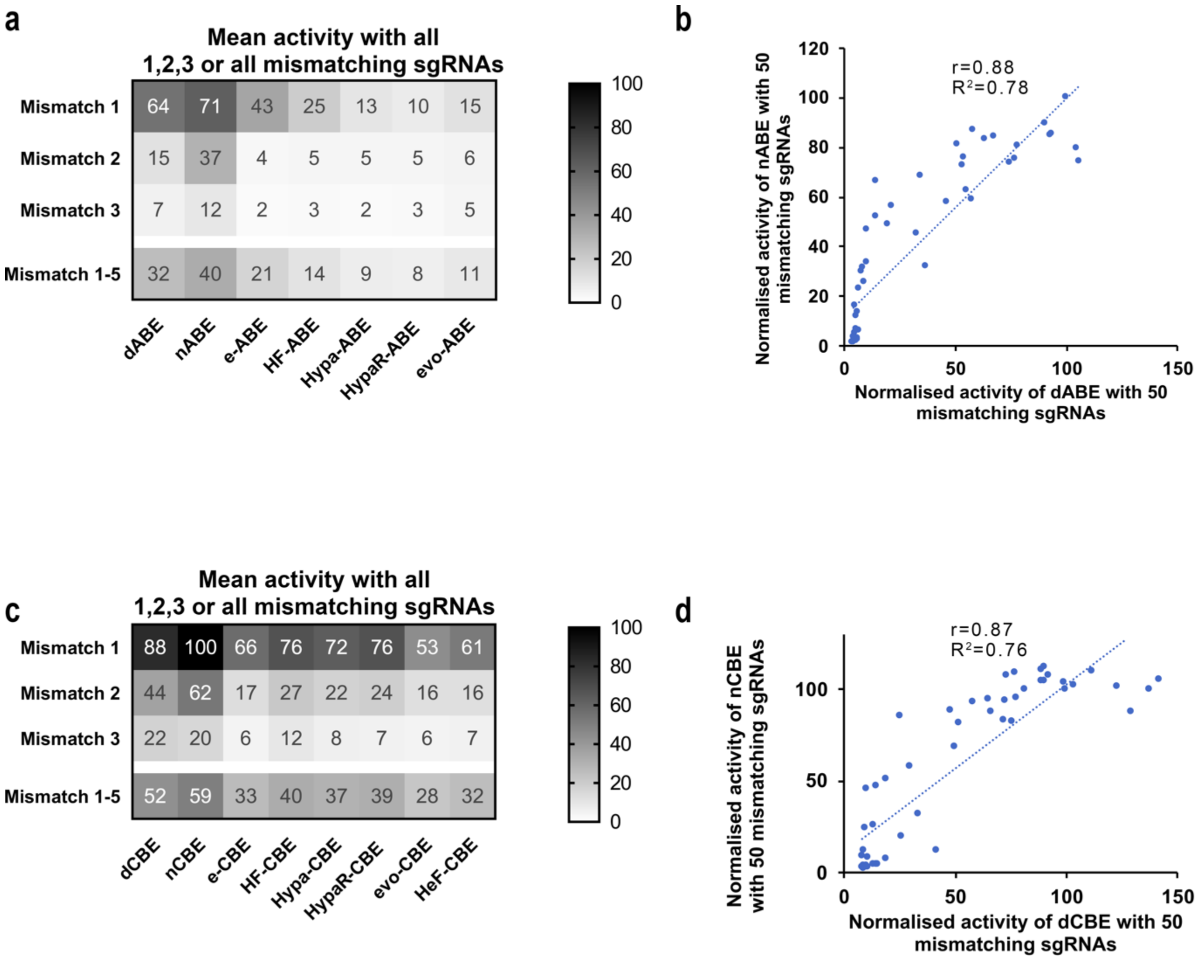

Supplementary Figure 6 – Activity of base editor variants with 50 mismatching sgRNAs

(**a**) Mean activity of different ABE variants with all 1, 2, 3 or all (1-5) mismatching sgRNAs that are shown in **Figure 6**.

(**b**) Scatter plot of editing activities of dABE and nABE on 50 mismatching sgRNAs that are shown on **Figure 6** (Pearson's r= 0.88).

(**c**) Mean activity of different CBE variants with all 1, 2, 3 or all (1-5) mismatching sgRNAs that are shown in **Figure 6**.

(**d**) Scatter plot of editing activities of dCBE and nCBE on 50 mismatching sgRNAs that are shown on **Figure 6** (Pearson's r= 0.87).

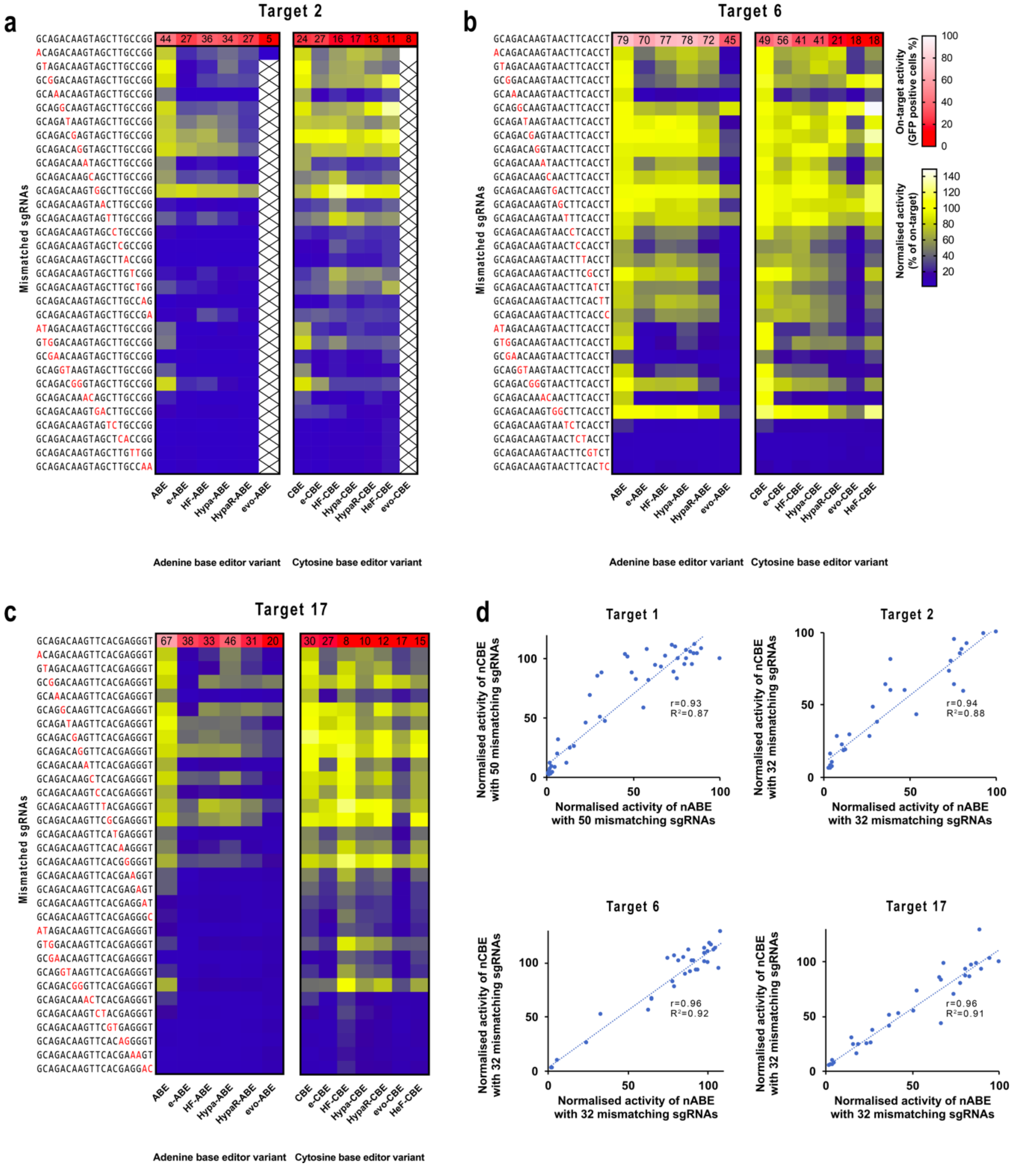

Supplementary Figure 7 – Off-target activities of different ABE variants on three additional targets with 1 matched and 31 mismatched sgRNAs

Mismatch tolerance of ABE and CBE and their high-fidelity variants were compared utilizing the same matching and 31 mismatching sgRNAs, the latter mismatching in either one or two positions, on targets 2 (**a**), 6 (**b**) and 17 (**c**). Blue-yellow heatmaps show the normalized activity (off-target/on-target) derived from three parallel transfections. White-red heatmaps show the on-target activity (mean rates of GFP positive cells) derived from three parallel transfections. For source data see Supplementary Data.

(**d**) Scatter plots of editing activities of nABE and nCBE on targets 1, 2, 6 and 17 on 50 and 32 mismatching sgRNAs that are shown on Figure 6 and on **a, b** and **c** (Pearson's r= 0.93, 0.94, 0.96 and 0.96, respectively).

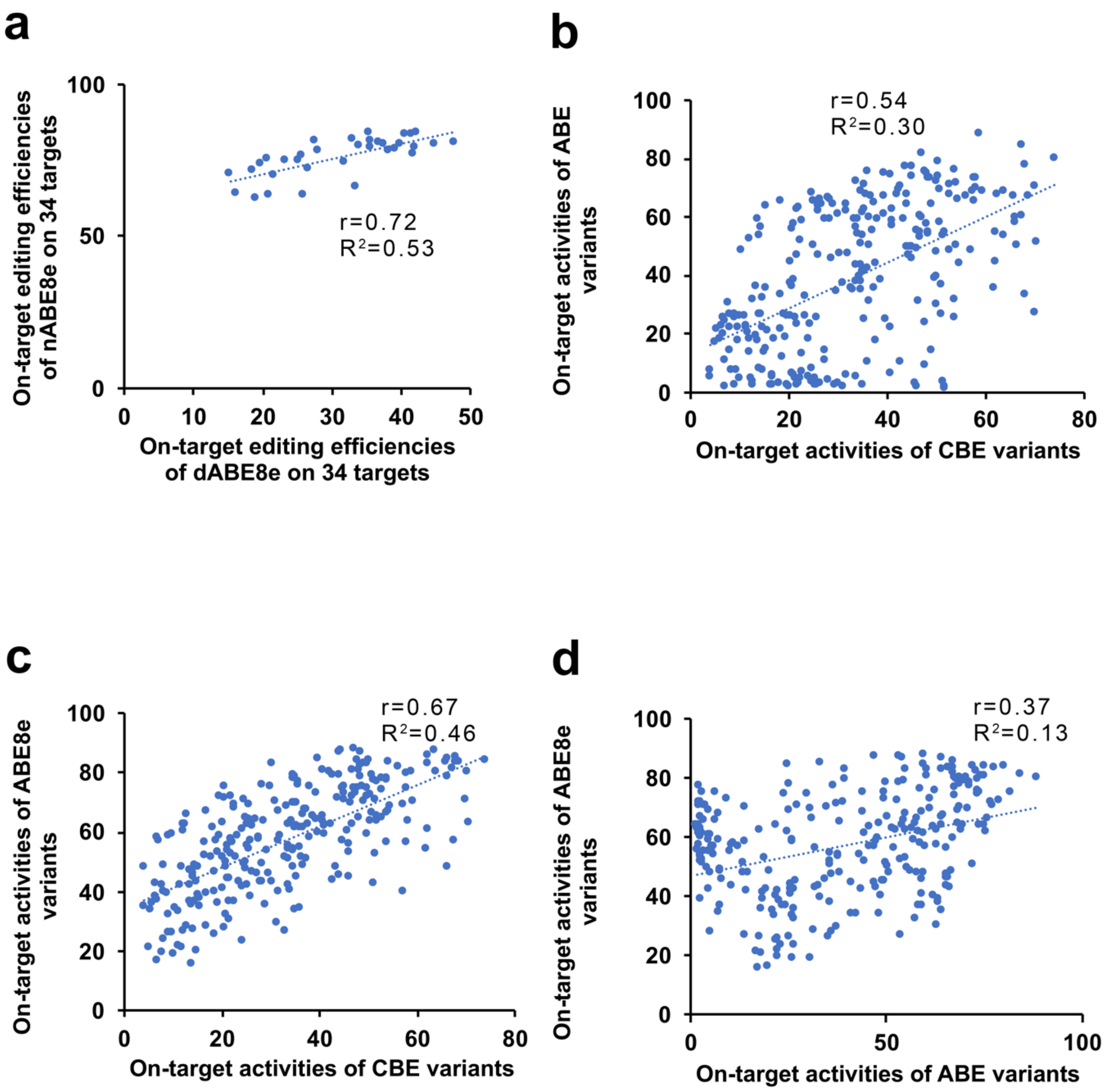

Supplementary Figure 8 – The activities of ABE8e variants are more similar to CBE than to ABE

(a) Scatter plot of the on-target editing efficiencies of on 34 target sites of nABE8e and dABE8e.

Scatter plot of the on-target editing efficiencies when either all (**b**) ABE and CBE variants (from Fig. 5); or (**c**) ABE8e and CBE variants (see Fig. 5 and Fig. 7); or (**d**) ABE8e and ABE variants (see Fig. 5 and Fig. 7) are compared on 34 target sites.

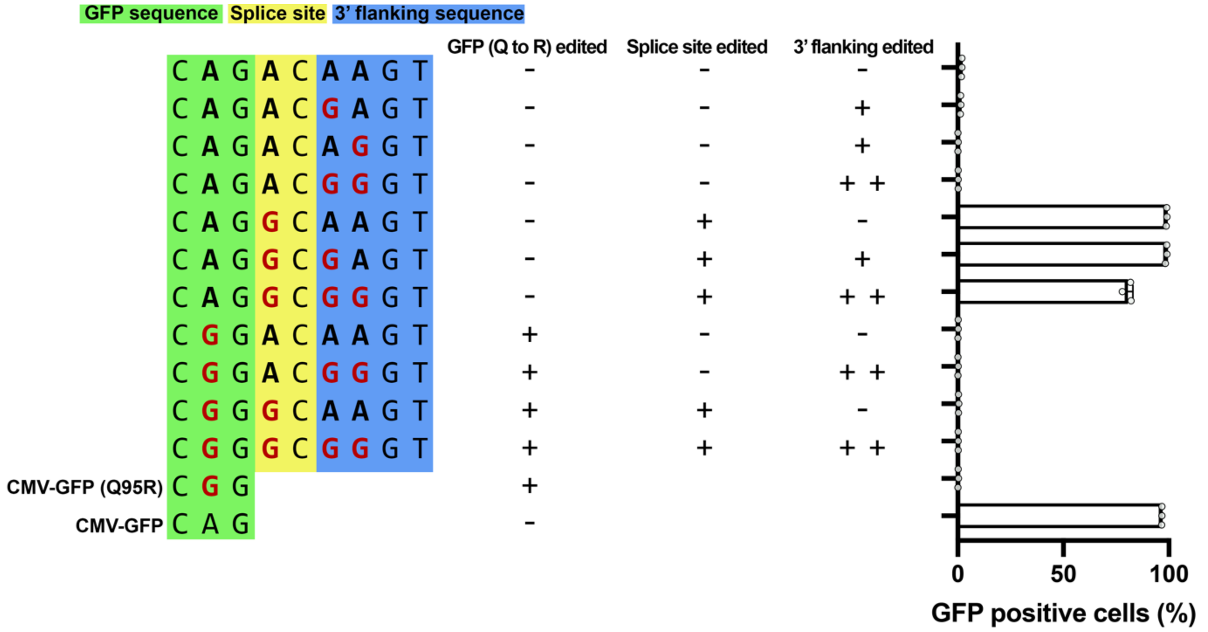

Supplementary Figure 9 – Bystander editing of ABE8e

Various splice donor sites (yellow) and their sequence context in the BEAR GFP plasmid representing potential bystander-edited sequences are shown on the left panel; the last amino acid of GFP exon1 is displayed in green and the 3' flanking sequence of the splice site in blue. On the right panel the percentages of GFP positive cells (measured by flow cytometry) are shown when transfected with various BEAR-GFP constructs (without base editors), where the adenines in the targets of the BEAR sequence are changed to guanines by molecular cloning. These modifications represent the bases which are modified by ABE8e. As controls, the last two constructs do not contain an intron, they express either a wild type GFP or a GFP with a modified amino acid (Q95R). Columns represent means +/- SD of three parallel transfections (grey circles).

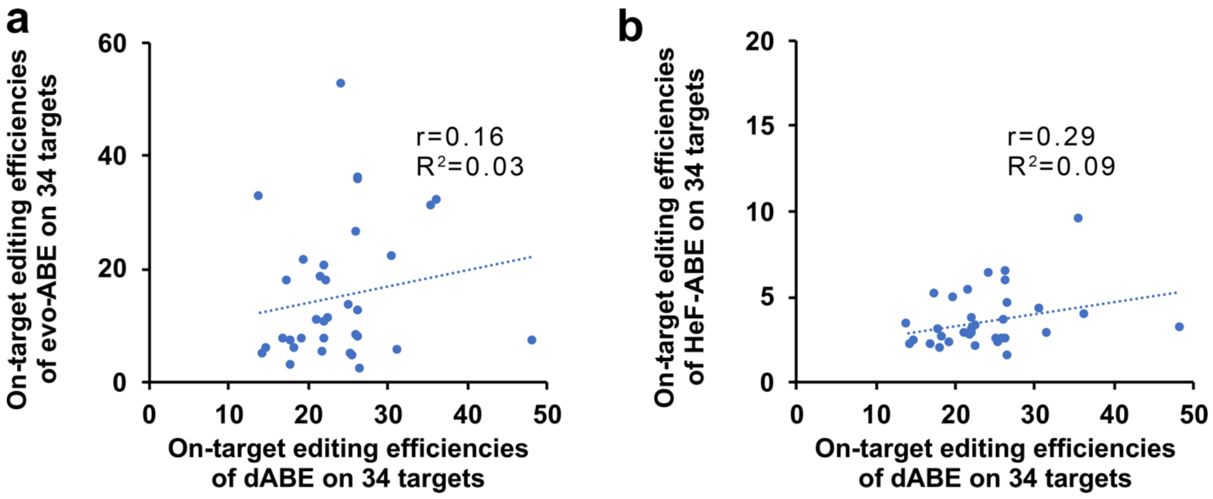

Supplementary Figure 10 – The on-target activity of dABE barely correlates with evo- or HeF-ABE

Scatter plot of the on-target editing efficiency of dABE, versus either (a) evo-ABE and (b) HeF-ABE on 34 target sites**.**

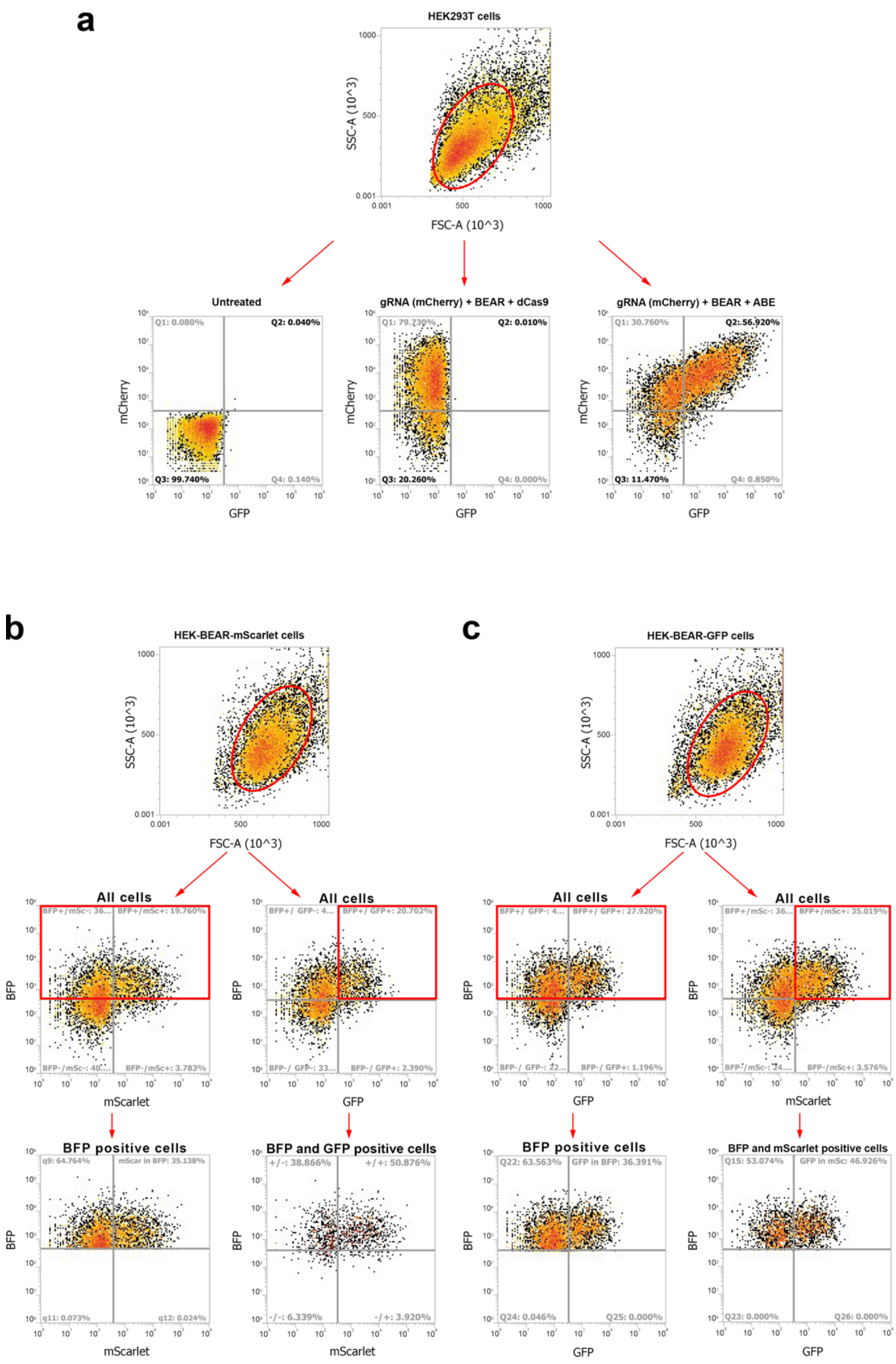

Supplementary Figure 11 – Flow cytometry gating examples

Flow cytometry gating examples are shown for different BEAR experiments. On panel (**a**) a density plot of HEK293T cells is shown when all live single cells (determined by FSC and SSC parameters) are either (1) untransfected (left plot) and thus show no fluorescence for mCherry or GFP, (2) co-transfected with an sgRNA-mCherry plasmid and the BEAR-GFP plasmid but with a dCas9, thus displaying mCherry but no GFP fluorescence, (3) or sgRNA-mCherry and BEAR-GFP plasmid is co-transfected with an ABE expressing plasmid, causing the splice site to be edited, and thus displaying both mCherry and GFP fluorescence. On panel (**b**) a gating example is shown from **Fig. 4a** where cells harbouring genomically integrated copies of BEAR-mScarlet sequences are co-edited alongside the

BEAR-GFP plasmid. On the upper left panel mScarlet positive cells are counted in all cells regardless of the presence of another fluorescent protein. The lower left panel shows mScarlet positive cells gated in the BFP positive population which accounts for the transfection marker enriched cells. On the upper right panel GFP and BFP positive cells are gated, and in this population mScarlet positive cells are counted (BEAR-GFP enrichment). On panel (**c**) a gating example is shown from **Fig. 4a** where cells harbouring genomically integrated copies of BEAR-GFP sequences are co-edited alongside the BEAR-mScarlet plasmid. On the upper left panel GFP positive cells are counted in all cells regardless of the presence of another fluorescent protein. The lower left panel shows GFP positive cells gated in the BFP positive population which accounts for the transfection marker enriched cells. On the upper right panel mScarlet and BFP positive cells are gated, and in this population GFP positive cells are counted (BEAR-mScarlet enrichment).

Supplementary Methods – Detailed plasmid construction

The sequences of oligos used to construct all plasmids are listed in Supplementary Table 1.

To construct BEAR-GFP plasmid candidates, a target cloning plasmid (pAT9624-BEAR-cloning) was cloned first, in which the target sequence is freely variable and can be cloned between the Esp3I sites via one-pot cloning (see below). The GFP halves were amplified via PCR from pEGFP-C1 (Clonthech) using primers *pAT9624-i1-for* and *pAT9624-i1-rev* and *pAT9624-i3-for* and *pAT9624-i3-rev*. The *Vim* intron sequence was amplified from the N2a genomic DNA with primers *pAT9624-i2-for* and *pAT9624-i2-rev*. The three inserts were cloned into an EcoRI and BshTI digested EGFP-C1 plasmid via Hi-Fi DNA Assembly.

For splice site screens and for on-target experiments all BEAR targets were cloned to pAT9624-BEAR-cloning between the Esp3I sites via one-pot cloning. Briefly, 2 units of Esp3I enzyme, 1.5 units of DNA ligase, 1 mM DTT, 500 μM ATP, 50 ng vector and 5-5 μM of target-coding oligonucleotides were mixed in Tango buffer, and the mixture was incubated at 37 °C for 30 minutes before being transformed into NEB5-alpha competent cells.

To clone sgRNA targets used with BEAR-GFP, an sgRNA cloning plasmid pAT9658-sgRNA-mCherry was constructed from an EF1-mCherry coding plasmid and a pU6-pegRNA-GG-acceptor (Addgene #132777) via Hi-Fi DNA Assembly. This plasmid expresses an mCherry protein in mammalian cells, which is applicable to monitor transfection efficiency, and an mRFP sequence in bacteria between the target cloning sites, which induces bacterial colonies to turn white instead of red upon successful cloning. The cloning site is BpiI, so all other enzyme recognition sites were mutated by introducing silent mutations into the mRFP1 plasmid. All sgRNA targets were cloned into this plasmid, to the BpiI sites, via one-pot cloning using the above described protocol with minute modifications (i.e. Green buffer and no DTT was used).

To clone the sgRNA targets used with BEAR-mScarlet and -mCherry, an sgRNA cloning plasmid pAT9679-BFP-sgRNA was constructed. mCherry in plasmid pAT9658-sgRNA-mCherry was replaced with BFP between BamHI and BglII sites via amplifying BFP by PCR, using primers *pAT9679-for* and *-rev*, and assembling the fragments via Hi-Fi DNA Assembly. This plasmid expresses a BFP protein in mammalian cells, which is applicable to monitor transfection efficiency, and can be used along with mScarlet and mCherry coding BEAR plasmids.

To construct the ABE coding pAT9676-ABE plasmid, the ABE coding sequence was amplified from pLenti-ABERA-P2A-Puro (Addgene #112675) using primers *pAT9676-ABE-for* and *-rev*, and it was cloned to the ZraI and PscI sites of a pUC19 plasmid via Hi-Fi DNA Assembly. A BGH polyA sequence was amplified via PCR with primers *pAT9676-BGH-for* and *-rev*, and it was cloned to the PscI site of the previous construct via Hi-Fi DNA Assembly.

To construct the dABE coding pAT9749-dABE plasmid from pAT9676-ABE, site-directed mutagenesis was applied to mutate the H840A amino acid, in order to gain a nuclease inactive ABE. pAT9676-ABE was digested with PscI and Eco32I. The respective discarded section of Cas9 was

amplified from the same plasmid, in two fragments, using primers *pAT9749-F1-for* and *-rev* and *pAT9749-F2-for* and *-rev*, in which the overlapping parts coded the mutant amino acid. The vector and the two fragments were assembled via Hi-Fi DNA Assembly.

To construct the CBE coding pAT9675-CBE plasmid, the CBE coding sequence was amplified from pLenti-FNLS-P2A-Puro (Addgene #110841) with primers *pAT9675-CBE-for* and *-rev*, and it was cloned to the ZraI and PscI sites of a pUC19 plasmid via Hi-Fi DNA Assembly. A BGH polyA sequence was amplified by PCR, using primers *pAT9675-BGH-for* and *pAT9676-BGH-rev*, and it was cloned to the PscI site of the previous construct via Hi-Fi DNA Assembly.

To construct the dCBE coding pAT9748-dCBE plasmid from pAT9675-CBE, site directed mutagenesis was applied to mutate the H840A amino acid, in order to gain a nuclease inactive CBE. pAT9675-CBE was digested with PscI and Eco32I. The respective discarded section of Cas9 was amplified from the same plasmid, in two fragments, with primers *pAT9749-F1-for* and *-rev* and *pAT9749-F2-for* and *-rev,* in which the overlapping parts coded the mutant amino acid. The vector and the two fragments were assembled via Hi-Fi DNA Assembly.

The Cas9 coding plasmid used was pX330-Flag-wtSpCas9 (Addgene #92353). The dCas9 coding plasmid used was pX330-Flag-dSpCas9 (Addgene #92113). The nCas9 coding plasmid used was pX330-Flag-wtSpCas9-D10A (Addgene #80448).

To construct the pAT9750-BEAR-mCherry plasmid with inactive splice donor site, the two mCherry halves were amplified from a pcDNA3.1-mCherry (Addgene #128744) plasmid via PCR using primers *pAT9750-i1-for* and *-rev* and *pAT9750-i3*-for and *-rev*. The *Vim* intron sequence was amplified from pAT9651-BEAR-GFP with primers *pAT9750-i2-for* and *pAT9750-i2-rev.* The three inserts were cloned into an EcoRI and BshTI digested EGFP-C1 plasmid via Hi-Fi DNA Assembly.

To construct the pAT9751-BEAR-mCherry-active plasmid with pre-edited splice donor site, the two mCherry halves were amplified from a pcDNA3.1-mCherry (Addgene #128744) plasmid via PCR using primers *pAT9751-i1-for* and *pAT9750-i1-rev* and *pAT9750-i3*-for and *-rev*. The *Vim* intron sequence was amplified from pAT9651-BEAR-GFP with primers *pAT9750-i2-for* and *pAT9750-i2-rev.* The three inserts were cloned into an EcoRI and BshTI digested EGFP-C1 plasmid via Hi-Fi DNA Assembly.

To construct the BEAR-mScarlet plasmids, mScarlet CDS from pCytERM_mScarlet_N1 (Addgene #85066) was cloned into pEGFP-C1 (pmScarlet-C1). To construct pAT9752-BEAR-mScarlet with inactive splice donor site, the two pmScarlet halves were amplified from the mScarlet-C1 plasmid via PCR using primers *pAT9624-i1-for* and *pAT9752-i1-rev* and *pAT9752-i3*-for and *pAT9624-i3-rev*. The *Vim* intron sequence was amplified from pAT9651-BEAR-GFP with primers *pAT9750-i2-for* and *pAT9752-i2-rev.* The three inserts were cloned into an EcoRI and BshTI digested EGFP-C1 plasmid via Hi-Fi DNA Assembly.

To construct pAT9753-BEAR-mScarlet with pre-edited splice donor site, the two mScarlet halves were amplified from the mScarlet-C1 plasmid via PCR using primers *pAT9624-i1-for* and *pAT9753-i1-rev* and *pAT9752-i3*-for and *pAT9624-i3-rev*. The *Vim* intron sequence was amplified from pAT9651-

BEAR-GFP with primers *pAT9750-i2-for* and *pAT9752-i2-rev.* The three inserts were cloned into an EcoRI and BshTI digested EGFP-C1 plasmid via Hi-Fi DNA Assembly.

To construct high fidelity ABE and CBE variants (pAT9991-eABE, pAT9992-HF-ABE, pAT9993-Hypa-ABE, pAT9994-HypaR661A-ABE, pAT9995-evoABE, pAT9996-HeF-ABE, pAT15064-eCBE, pAT15065-HF-CBE, pAT15066-Hypa-CBE, pAT15067-HypaR661A-CBE, pAT15068-evoCBE, pAT15069-HeF-CBE) increased fidelity Cas9 coding sequences (pX330-Flag-eSpCas9, -SpCas9-HF1, -HypaSpCas9, -Hypa-A-SpCas9, -evoSpCas9, -HeFSpCas9 – Addgene #126754-126459) were amplified by PCR using primers *pAT9991-for* and *pAT9749-F2-rev.* To construct the ABE variants, the amplified increased fidelity Cas9 fragments were cloned to a PScI and BglII digested pAT9676-ABE plasmid via Hi-Fi DNA Assembly. To construct the CBE variants, the amplified increased fidelity Cas9 fragments were cloned to a PScI and BglII digested pAT9675-CBE plasmid via Hi-Fi DNA Assembly. To append the UGI sequence to the CBE variants, high fidelity variant constructs and pAT9675-CBE were digested with NotI and Mva1269I, respectively and the UGI containing fragment was ligated with a T4 ligase.

To construct the editing window screen target plasmids with pre-edited or inactive splice donor site, GFP halves were amplified from the EGFP-C1 plasmid with primers listed in the primer list below (insert 1 and insert 3), and the intron sequence (insert 2) was amplified from the pAT9651-BEAR-GFP plasmid with primers listed in the primer list below. The three inserts were cloned into an EcoRI and BshTI digested EGFP-C1 plasmid via Hi-Fi DNA Assembly. All sgRNA targets were cloned to the plasmid pAT9658-sgRNA-mCherry plasmid using the protocol described above.

To construct BEAR-GFP and BEAR-mScarlet plasmids with different intron positions, GFP and mScarlet halves were amplified from pEGFP-C1 and mScarlet-C1 plasmids via PCR, using "insert 1 and insert 3" primers, as indicated in Supplementary Table 1 under the title "BEAR plasmids with different intron positions". The *Vim* intron sequence was amplified from the pAT9651-BEAR-GFP plasmid via PCR, using "insert 2" primers.

To construct BEAR-GFP and BEAR-mScarlet expressing cell lines, the pSc1-puro (Addgene #80438) plasmid was modified to eliminate the sgRNA coding sequence, but not its target site. The GFP sequence was replaced by the coding sequences of BEAR-GFP and BEAR-mScarlet, which were cloned to the BshTI and EcoRI sites of this plasmid. The spacer that targets and linearizes these plasmids within the cells was cloned to pmCherry-gRNA (Addgene #80457) using the above described one-pot cloning method, utilizing oligos *U6-TL oligo-1* and *-2.* AAVS1 targeting spacers (AAVS1-a and -b) were cloned to pmCherry-gRNA (Addgene #80457) using the above described one-pot cloning method, utilizing oligos *AAVS1-a* and *-b, oligo-1 and -2.* U6-AAVS1-a was used to construct BEAR-GFP, and U6-AAVS1-b was used to create BEAR-mScarlet cell lines.

To construct pAT15516_BEAR-GFP-2in1 the coding sequence of BEAR-GFP was amplified by PCR from pAT9651 using primers *BEAR-GFP-2in1-for and -rev* and the product was cloned into a MunI and NotI digested pAT15415 plasmid via HiFi Assembly.

For the enrichment experiments genomic targets were cloned to pAT9679-BFP-sgRNA using the above described one-pot cloning method, utilizing oligos listed in Table 2 (section - *oligonucleotides used for cloning genomic sgRNA targets*).

For the on-target experiments, sgRNA spacers (T1-T34) were cloned into the plasmid pAT9658-sgRNA-mCherry plasmid.

For the off-target experiments, mismatching sgRNA spacers (targets: T1, T2, T6, T7 and T17) were cloned into the plasmid pAT9658-sgRNA-mCherry plasmid.

To construct ABE8e variants ABE8e sequence was amplified from plasmid ABE8e (Addgene #138489) using primers (*ABE8e-for and ABE8e-rev)* and the product was cloned into NcoI and Eco81I digested ABE coding plasmids (pAT9676, pAT9749, pAT9991, pAT9992, pAT9993, pAT9994, pAT9995, pAT9996) via HiFi Assembly.

Method	Base editor used				Is the reporter transient?	Background signal	Number of targets tested	Number of possible targets	Can CBE and ABE be compared on the same target sequences?	Is enrichment with transient reporter demonstrated?	Can indels generate signal?
	nCBE	nABE	dCBE	dABE							
**BE-FLARE**	yes	no	no	no	yes	<0.5 %	1	1	no	yes (FACS)	ND
**ACE**	yes	no	no	no	yes	0-6%	1	1	no	no*	8% background signal with nCas9, 70% with Cas9
**GFP panel**	yes	no	no	no	yes	<0.5 %	3	restricted	no	no	ND
**TREE**	yes	no	no	no	yes	<0.5 %	1	1	no	yes (FACS)	ND
**GO**	yes	yes	no	no	ND	<0.5 %	10	restricted	no	no*	Not sensitive
**BEON**	no	yes	no	no	yes	5-20%	14	minimaly restricted	no	yes (FACS)	ND
**BEAR (this study)**	yes	yes	yes	yes	yes	<0.5 %	79	minimaly restricted	yes	yes (FACS)	Not sensitive

Supplementary Table 1 – Comparison of seven fluorescence-based markers of base editing

Seven fluorescent markers of base editing are compared (including this work). In the first column we compared whether the assay was demonstrated on detecting CBE or ABE using a nickase (nCBE, nABE) or nuclease inactive (dCBE, dABE) Cas9 partner. In the next column we compare if the method was used with a transient (plasmid) reporter. Next, we evaluate the amount of background fluorescent signal that is produced by the markers in a negative control condition (fluorescent marker alone without base editors). Next, the actual number of tested and the theoretically possible target sites are counted. Next, we compare whether the assay has the possibility of testing CBE and ABE on the exact same spacer sequences. After that, we indicate whether any base editing enrichment was demonstrated and if so, then whether it was with a transient or with a genomically integrated reporter. In the last column we reveal if any experiments were shown to detect the amount of generated fluorescent signal when the reporter is targeted by a nickase or a nuclease Cas9. "ND" abbreviation in the table means that that specific feature was not demonstrated in the publication. "*" abbreviation in the table means that enrichment was demonstrated in the publication but only on a genomically integrated reporter, not on a transient one.

Supplementary Table 2 – List of oligonucleotides

**Oligonucleotides used for BEAR-GFP target cloning plasmid pAT9624**		
**oligo name**	**oligo sequence**	
pAT9624-i1-for	GTGAACCGTCAGATCCGCTAG	
pAT9624-i1-rev	TGAGACGTAAGATCTCCTCGTCTCGCACGTAGCCTTCGGGCATG	
pAT9624-i2-for	GAGACGAGGAGATCTTACGTCTCAATTTTTAGTTAAAATATGGGAAAG	
pAT9624-i2-rev	CGTCCTTGAAGAAGATGGTGCGCTCCTGCTCAAAAAAGAAAC	
pAT9624-i3-for	GAGCGCACCATCTTCTTCAAGGACG	
pAT9624-i3-rev	CCCGCGGTACCGTCGAC	
**Oligonucleotides used for cloning splice site screen target plasmids (Fig. 2, Supplementary Fig.2)**		
**plasmid name**	**oligo1**	**oligo2**
**P1 - active**	CGTGCAGGCAAGTGCATAGACTGCGGGTTG	AAATCAACCCGCAGTCTATGCACTTGCCTG
**P1 - inactive**	CGTGCAGACAAGTGCATAGACTGCGGGTTG	AAATCAACCCGCAGTCTATGCACTTGTCTG
**P2 - active**	CGTGCAAGTAAGTGCATAGACTGCGGGTTG	AAATCAACCCGCAGTCTATGCACTTACTTG
**P2 - inactive**	CGTGCAAATAAGTGCATAGACTGCGGGTTG	AAATCAACCCGCAGTCTATGCACTTATTTG
**P3 - active**	CGTGCAGGTTGAGGCATAGACTGCGGGTTG	AAATCAACCCGCAGTCTATGCCTCAACCTG
**P3 - inactive**	CGTGCAGATTGAGGCATAGACTGCGGGTTG	AAATCAACCCGCAGTCTATGCCTCAATCTG
**P4 - active**	CGTGCAGTTAAGTGCTGGAGGTGGGGGTTG	AAATCAACCCCCACCTCCAGCACTTAACTG
**P4 - inactive**	CGTGCAGTCAAGTGCTGGAGGTGGGGGTTG	AAATCAACCCCCACCTCCAGCACTTGACTG
**P5 - active**	CGTGCAGGTTGCGGCTGGAGGTGGGGGTTG	AAATCAACCCCCACCTCCAGCCGCAACCTG
**P5 - inactive**	CGTGCAGGCTGCGGCTGGAGGTGGGGGTTG	AAATCAACCCCCACCTCCAGCCGCAGCCTG
**Oligonucleotides used for cloning sgRNA targets on Fig. 2c, d**		
**plasmid name**	**oligo1**	**oligo2**
**P1-sgRNA**	CACCGCAGACAAGTGCATAGACTG	AAACCAGTCTATGCACTTGTCTGC
**P2-sgRNA**	CACCGCAAATAAGTGCATAGACTG	AAACCAGTCTATGCACTTATTTGC
**P3-sgRNA**	CACCGCAGATTGAGGCATAGACTG	AAACCAGTCTATGCCTCAATCTGC
**P4-sgRNA**	CACCGCAGTCAAGTGCTGGAGGTG	AAACCACCTCCAGCACTTGACTGC
**P5-sgRNA**	CACCGCAGGCTGCGGCTGGAGGTG	AAACCACCTCCAGCCGCAGCCTGC

**Oligonucleotides used for cloning ABE, ABE8e, CBE and their increased fidelity variants, Fig. 2-6**		
**oligo name**	**oligo sequence**	
pAT9676-ABE-for	ATTTCCCCGAAAAGTGCCACCTGACGTCCAGCAGAGATCCACTTTGG	
pAT9676-ABE-rev	TGGCCTTTTGCTGGCCTTTTGCTCACATGTCATTTCTTTTTCTTAGCTTGACCAG	
pAT9676-BGH-for	GCTAAGAAAAAGAAATGACATGTCCTAGAGCTCGCTGATCAGCCTCG	
pAT9676-BGH-rev	TTTTGCTGGCCTTTTGCTCAGCGGCCGCTCCCCAG	
pAT9749-F1-for	GAGGAAAACGAGGACATTCTGGAAGAT	
pAT9749-F1-rev	GAAAGCTCTGAGGCACGATGGCGTCCACATCGTAGTCGG	
pAT9749-F2-for	CCGACTACGATGTGGACGCCATCGTGCCTCAGAGCTTTC	
pAT9749-F2-rev	GGCTGATCAGCGAGCTCTAGG	
pAT9675-CBE-for	ATTTCCCCGAAAAGTGCCACCTGACGTCCAGCAGAGATCCACTTTGG	
pAT9675-CBE-rev	TGGCCTTTTGCTGGCCTTTTGCTCACATGTCAGACTTTCCTCTTCTTCTTGG	
pAT9675-BGH-for	AGAAGAAGAGGAAAGTCTGACATGTCCTAGAGCTCGCTGATCAGCCTCG	
pAT9991-for	GAACCGGATCTGCTATCTGCAAGA	
ABE8e-for	cgtgacgcgggatccgccaccatgaaacg	
ABE8e-rev	gaccccccagagctaccacctgaggattcaggtgttgcgctctcg	
**Oligonucleotides used for cloning editing window BEAR target plasmids (Sup. Fig. 3a)**		
**oligo name**	**insert1 - fwd oligo**	
Window - inactive - A20-1-i1-for	GTGAACCGTCAGATCCGCTAG	
Window - inactive - A20-19-i1-rev	CCGCAGTCTATGCCACACCCATCAGGGCACGGGCAG	
Window - inactive - A20-1-i2-rev	GGGTGGTCACGAGGGTGGGCCTGCTCAAAAAAGAAAC	
Window - inactive - A20-1-i3-for	GCCCACCCTCGTGACCAC	
Window - inactive - A20-1-i3-rev	CCCGCGGTACCGTCGAC	
Window - inactive - A20-19-i1-rev	CCGCAGTCTATGCCACACCCATCAGGGCACGGGCAG	
Window - inactive - A20-19-i2-for	GGGTGTGGCATAGACTGCGGG	
Window - inactive - A18-i2-for	CAAGCTGCCCGTGCCCTGATGGGTTGGCATAGACTGCGGG	
Window - inactive - A17-i2-for	CAAGCTGCCCGTGCCCTGATGGGTGGCATAGACTGCGGG	
Window - inactive - A16-i2-for	CAAGCTGCCCGTGCCCTGATGGGTGCATAGACTGCGGG	
Window - inactive - A15-i2-for	CAAGCTGCCCGTGCCCTGATGGGTCATAGACTGCGGGTTG	
Window - inactive - A14-i2-for	CAAGCTGCCCGTGCCCTGATGGGTATAGACTGCGGGTTG	
Window - inactive - A13-i2-for	CAAGCTGCCCGTGCCCTGATGGGTTAGACTGCGGGTTG	
Window - inactive - A12-i2-for	CAAGCTGCCCGTGCCCTGATGGGTAGACTGCGGGTTG	

Window - inactive - A11-i2-for	CAAGCTGCCCGTGCCCTGATGGGTGACTGCGGGTTGA	
Window - inactive - A10-i2-for	CAAGCTGCCCGTGCCCTGATGGGTACTGCGGGTTGATTTTTTAG	
Window - inactive - A9-i2-for	CAAGCTGCCCGTGCCCTGATGGGTCTGCGGGTTGATTTTTTAG	
Window - inactive - A8-i2-for	CAAGCTGCCCGTGCCCTGATGGGTGCGGGTTGATTTTTTAG	
Window - inactive - A7-i2-for	CAAGCTGCCCGTGCCCTGATGGGTCGGGTTGATTTTTTAG	
Window - inactive - A6-i2-for	CAAGCTGCCCGTGCCCTGATGGGTGGGTTGATTTTTTAGTTAAAATATG	
Window - inactive - A5-1-i2-for	CAAGCTGCCCGTGCCCTGATGGGGGGTTGATTTTTTAGTTAAAATATG	
Window - active 20-1-i1-for	GTGAACCGTCAGATCCGCTAG	
Window - active 20-1-i2-rev	GGGTGGTCACGAGGGTGGGCCTGCTCAAAAAAGAAAC	
Window - active 20-1-i3-for	GCCCACCCTCGTGACCAC	
Window - active 20-1-i3-rev	CCCGCGGTACCGTCGAC	
Window - active 20-19-i1-rev	CCGCAGTCTATGCCACACCCACCAGGGCACGGGCAG	
Window - active 18-1-i1-rev	ACCAGGGCACGGGCAG	
Window - active 20-19-i2-rev	GGGTGTGGCATAGACTGCGGG	
Window - active 18-i2-rev	CAAGCTGCCCGTGCCCTGGTGGGTTGGCATAGACTGCGGG	
Window - active 17-i2-rev	CAAGCTGCCCGTGCCCTGGTGGGTGGCATAGACTGCGGG	
Window - active 16-i2-rev	CAAGCTGCCCGTGCCCTGGTGGGTGCATAGACTGCGGG	
Window - active 15-i2-rev	CAAGCTGCCCGTGCCCTGGTGGGTCATAGACTGCGGGTTG	
Window - active 14-i2-rev	CAAGCTGCCCGTGCCCTGGTGGGTATAGACTGCGGGTTG	
Window - active 13-i2-rev	CAAGCTGCCCGTGCCCTGGTGGGTTAGACTGCGGGTTG	
Window - active 12-i2-rev	CAAGCTGCCCGTGCCCTGGTGGGTAGACTGCGGGTTG	
Window - active 11-i2-rev	CAAGCTGCCCGTGCCCTGGTGGGTGACTGCGGGTTGA	
Window - active 10-i2-rev	CAAGCTGCCCGTGCCCTGGTGGGTACTGCGGGTTGATTTTTTAG	
Window - active 9-i2-rev	CAAGCTGCCCGTGCCCTGGTGGGTCTGCGGGTTGATTTTTTAG	
Window - active 8-i2-rev	CAAGCTGCCCGTGCCCTGGTGGGTGCGGGTTGATTTTTTAG	
Window - active 7-i2-rev	CAAGCTGCCCGTGCCCTGGTGGGTCGGGTTGATTTTTTAG	
Window - active 6-i2-rev	CAAGCTGCCCGTGCCCTGGTGGGTGGGTTGATTTTTTAGTTAAAATATG	
Window - active 5-1-i2-rev	CAAGCTGCCCGTGCCCTGGTGGGGGGTTGATTTTTTAGTTAAAATATG	
**Oligonucleotides used for cloning editing window sgRNA targets (Sup. Fig. 3a)**		
**plasmid name**	**oligo1**	**oligo2**
sgRNA - window - 20	CACCATGGGTGTGGCATAGACTGC	AAACGCAGTCTATGCCACACCCAT
sgRNA - window - 19	CACCGATGGGTGTGGCATAGACTG	AAACCAGTCTATGCCACACCCATC
sgRNA - window - 18	CACCGTGATGGGTTGGCATAGACTG	AAACCAGTCTATGCCAACCCATCAC

sgRNA - window - 17	CACCGCTGATGGGTGGCATAGACTG	AAACCAGTCTATGCCACCCATCAGC
sgRNA - window - 16	CACCGCCTGATGGGTGCATAGACTG	AAACCAGTCTATGCACCCATCAGGC
sgRNA - window - 15	CACCGCCCTGATGGGTCATAGACTG	AAACCAGTCTATGACCCATCAGGGC
sgRNA - window - 14	CACCGCCCTGATGGGTATAGACTG	AAACCAGTCTATACCCATCAGGGC
sgRNA - window - 13	CACCGTGCCCTGATGGGTTAGACTG	AAACCAGTCTAACCCATCAGGGCAC
sgRNA - window - 12	CACCGGTGCCCTGATGGGTAGACTG	AAACCAGTCTACCCATCAGGGCACC
sgRNA - window - 11	CACCGCGTGCCCTGATGGGTGACTG	AAACCAGTCACCCATCAGGGCACGC
sgRNA - window - 10	CACCGCCGTGCCCTGATGGGTACTG	AAACCAGTACCCATCAGGGCACGGC
sgRNA - window - 9	CACCGCCCGTGCCCTGATGGGTCTG	AAACCAGACCCATCAGGGCACGGGC
sgRNA - window - 8	CACCGCCCGTGCCCTGATGGGTGC	AAACGCACCCATCAGGGCACGGGC
sgRNA - window - 7	CACCGTGCCCGTGCCCTGATGGGTC	AAACGACCCATCAGGGCACGGGCAC
sgRNA - window - 6	CACCGCTGCCCGTGCCCTGATGGGT	AAACACCCATCAGGGCACGGGCAGC
sgRNA - window - 5	CACCGCTGCCCGTGCCCTGATGGG	AAACCCCATCAGGGCACGGGCAGC
sgRNA - window - 4	CACCAGCTGCCCGTGCCCTGATGG	AAACCCATCAGGGCACGGGCAGCT
sgRNA - window - 3	CACCAAGCTGCCCGTGCCCTGATG	AAACCATCAGGGCACGGGCAGCTT
sgRNA - window - 2	CACCGCAAGCTGCCCGTGCCCTGAT	AAACATCAGGGCACGGGCAGCTTGC
sgRNA - window - 1	CACCGCAAGCTGCCCGTGCCCTGA	AAACTCAGGGCACGGGCAGCTTGC
**Oligonucleotides used for cloning BEAR-mScarlet and BEAR-mCherry plasmids (Fig. 3b)**		
**oligo name**	**oligo sequence**	
pAT9750-i1-for	CGTCAGATCCGCTAGCGCTACCGGTCGCCACCATGGTGAGCAAG	
pAT9750-i1-rev	ACCCGCAGTCTATGCACTTGTCTGCAGGGAGGAGTCC	
pAT9750-i2-for	CAAGTGCATAGACTGC	
pAT9750-i2-rev	ACTCGCCGTCCTGCTCAAAAAAGAAAC	
pAT9750-i3-for	TTTTGAGCAGGACGGCGAGTTC	
pAT9750-i3-rev	CGCGGTACCGTCGACTGCAGCCCTCTAGATGCATGCTCG	
pAT9751-i1-rev	ACCCGCAGTCTATGCACTTGCCTGCAGGGAGGAGTCC	
pAT9752-i1-rev	ACCCGCAGTCTATGCACTTGTCTGCGTCACGGTCACGGCG	
pAT9752-i2-rev	AGGGAGGTGTCCTGCTCAAAAAAGAAAC	
pAT9752-i3-for	TTTTGAGCAGGACACCTCCCTGGAGG	
pAT9753-i1-rev	ACCCGCAGTCTATGCACTTGCCTGCGTCACGGTCACGGCG	
**Oligonucleotides used for cloning pAT9679-BFP-sgRNA (Fig. 3b)**		

**oligo name**	**oligo sequence**	
pAT9679-for	ACACAGGTGTCGTGACGCGGGATCCGCCACCATGAGCG	
pAT9679-rev	AGCGAGCTCTAGGACATGTAGATCTTAATTAAGCTTGTGCCCCAG	
**BEAR plasmids with different intron positions (Fig. 3c, d)**		
**oligo name**	**oligo sequence**	
all intron constructs - i1-for	GTGAACCGTCAGATCCGCTAG	
all intron constructs - i3-rev	CCCGCGGTACCGTCGAC	
GFP 53-i1-rev	CTGTGGTGCAGATAAACTTCAGGGTCAGCTTGCC	
GFP 53-i2-for	GAAGTTTATCTGCACCACAGGCAAGTGCATAGACTGCG	
GFP 53-i2-rev	GGCAGCTTGCCTGCTCAAAAAAGAAAC	
GFP 53-i3-for	TTTTGAGCAGGCAAGCTGCCCGTGCC	
GFP 58-i1-rev	ATCAGGGCACGGGCAG	
GFP 58-i2-for	CAAGCTGCCCGTGCCCTGATGGGTATAGACTGCGGGTTG	
GFP 58-i2-rev	GGGTGGTCACGAGGGTGGGCCTGCTCAAAAAAGAAAC	
GFP 58-i3-for	GCCCACCCTCGTGACCAC	
GFP 87-i1-rev	CTGACTTGAAGAAATCGTGCTGCTTCATG	
GFP 87-i2-for	GCACGATTTCTTCAAGTCAGGCAAGTGCATAGACTGCG	
GFP 87-i2-rev	CCTTCGGGCATGGCTGCTCAAAAAAGAAAC	
GFP 87-i3-for	TGAGCAGCCATGCCCGAAGGCTACG	
GFP 117-i1-rev	CCGCAGTCTATGCCACACCCACCCTCAAACTTCACCTCGGCGCG	
GFP 117-i2-for	GGGTGTGGCATAGACTGCGGG	
GFP 117-i2-rev	CGGTTCACCAGGGTGTCGCCTGCTCAAAAAAGAAAC	
GFP 117-i3-for	GCGACACCCTGGTGAACCG	
mScarlet 65-i1-rev	CTGAGGGGAAAGGATGTCCCAGGAGAAGGG	
mScarlet 65-i2-for	GGGACATCCTTTCCCCTCAGGCAAGTGCATAGACTGCG	
mScarlet 65-i2-rev	CGTACATGAACTGCTCAAAAAAGAAAC	
mScarlet 65-i3-for	TTTTGAGCAGTTCATGTACGGCTCCAGGG	
mScarlet 71-i1-rev	CTGGAGCCGTACATAAACTGAGGGGACAGGATG	
mScarlet 71-i2-for	CAGTTTATGTACGGCTCCAGGCAAGTGCATAGACTGCG	
mScarlet 71-i2-rev	GGTGAAGGCCCTGCTCAAAAAAGAAAC	
mScarlet 71-i3-for	TTTTGAGCAGGGCCTTCACCAAGCACC	
mScarlet 78-i1-rev	CTGGGTGCTTGGTAAAGGCCCTGGAGCCGTA	
mScarlet 78-i2-for	GGCCTTTACCAAGCACCCAGGCAAGTGCATAGACTGCG	
mScarlet 78-i2-rev	GGGATGTCGGCTGCTCAAAAAAGAAAC	

mScarlet 78-i3-for	TTTTGAGCAGCCGACATCCCCGACTACTATAAGCAG	
mScarlet 110-i1-rev	ACCCGCAGTCTATGCACTTGCCTGCAGGGAGGAGTCC	
mScarlet 110-i2-for	CAAGTGCATAGACTGC	
mScarlet 110-i2-rev	ACTCGCCGTCCTGCTCAAAAAAGAAAC	
mScarlet 110-i3-for	TTTTGAGCAGGACGGCGAGTTC	
**Oligonucleotides used for plasmids used in creating BEAR cell lines (Supplementary Fig.3, Fig. 4)**		
**plasmid name**	**oligo1**	**oligo2**
**U6-TL**	CACCGGCGCAACGCGATCGCGTAA	AAACTTACGCGATCGCGTTGCGCC
**U6-AAVS-1-a**	CACCACAGTGGGGCCACTAGGGAC	AAACGTCCCTAGTGGCCCCACTGT
**U6-AAVS-1-b**	CACCGGTCCCTAGTGGCCCCACTG	AAACCAGTGGGGCCACTAGGGACC
**Oligonucleotides used for cloning genomic sgRNA targets (Fig. 4)**		
**plasmid name**	**oligo1**	**oligo2**
**sgRNA HEK site 1**	CACCGGGAAAGACCCAGCATCCGT	AAACACGGATGCTGGGTCTTTCCC
**sgRNA HEK site 2**	CACCGAACACAAAGCATAGACTGC	AAACGCAGTCTATGCTTTGTGTTC
**sgRNA HEK site 3**	CACCGGCCCAGACTGAGCACGTGA	AAACTCACGTGCTCAGTCTGGGCC
**sgRNA HEK site 4**	CACCGGCACTGCGGCTGGAGGTGG	AAACCCACCTCCAGCCGCAGTGCC
**sgRNA CCR5**	CACCGGTACCTATCGATTGTCAGG	AAACCCTGACAATCGATAGGTACC
**sgRNA FANCF site 2**	CACCGCTGCAGAAGGGATTCCATG	AAACCATGGAATCCCTTCTGCAGC
**sgRNA SCN5a**	CACCGTTGCACAGAAGGGTAGGCA	AAACTGCCTACCCTTCTGTGCAAC
**Oligonucleotides used for cloning on-target screen, target plasmids (Fig. 5)**		
**plasmid name**	**oligo1**	**oligo2**
**On-target T1**	see "p1 inactive" cloning, above	
**On-target T2**	CGTGCAGACAAGTAGCTTGCCGGTGG	AAATCCACCGGCAAGCTACTTGTCTG
**On-target T3**	CGTGCAGACAAGTGCGACGTAAACGG	AAATCCGTTTACGTCGCACTTGTCTG
**On-target T4**	CGTGCAGACAAGTATGAACTTCAGGG	AAATCCCTGAAGTTCATACTTGTCTG
**On-target T5**	CGTGCAGACAAGTATCTTCTTCAAGG	AAATCCTTGAAGAAGATACTTGTCTG
**On-target T6**	CGTGCAGACAAGTAACTTCACCTCGG	AAATCCGAGGTGAAGTTACTTGTCTG
**On-target T7**	CGTGCAGACAAGTATGGTCCTGCTGG	AAATCCAGCAGGACCATACTTGTCTG

**On-target T8**	CGTGCAGACAAGTAGAGTGATCCCGG	AAATCCGGGATCACTCTACTTGTCTG
**On-target T9**	CGTGCAGACAAGTTTGAAGAAGATGG	AAATCCATCTTCTTCAAACTTGTCTG
**On-target T10**	CGTGCAGACAAGTGGCGACACCCTGG	AAATCCAGGGTGTCGCCACTTGTCTG
**On-target T11**	CGTGCAGACAAGTAGGGCGGACTGGG	AAATCCCAGTCCGCCCTACTTGTCTG
**On-target T12**	CGTGCAGACAAGTAGTGGTTGTCGGG	AAATCCCGACAACCACTACTTGTCTG
**On-target T13**	CGTGCAGACAAGTTCGCCCTCGCCGG	AAATCCGGCGAGGGCGAACTTGTCTG
**On-target T14**	CGTGCAGACAAGTATGCCACCTACGG	AAATCCGTAGGTGGCATACTTGTCTG
**On-target T15**	CGTGCAGACAAGTTGCACGCCGTAGG	AAATCCTACGGCGTGCAACTTGTCTG
**On-target T16**	CGTGCAGACAAGTTCGAGCTGAAGGG	AAATCCCTTCAGCTCGAACTTGTCTG
**On-target T17**	CGTGCAGACAAGTTCACGAGGGTGGG	AAATCCCACCCTCGTGAACTTGTCTG
**On-target T18**	CGTGCAGACAAGTTCGGGCATGGCGG	AAATCCGCCATGCCCGAACTTGTCTG
**On-target T19**	CGTGCAGACAAGTTCAAGGAGGACGG	AAATCCGTCCTCCTTGAACTTGTCTG
**On-target T20**	CGTGCAGACAAGTTGGTAGTGGTCGG	AAATCCGACCACTACCAACTTGTCTG
**On-target T21**	CGTGCAGACAAGTGGGTGGGCCAGGG	AAATCCCTGGCCCACCCACTTGTCTG
**On-target T22**	CGTGCAGACAAGTCTGTTCACCGGGG	AAATCCCCGGTGAACAGACTTGTCTG
**On-target T23**	CGTGCAGACAAGTGGCACCACCCCGG	AAATCCGGGGTGGTGCCACTTGTCTG
**On-target T24**	CGTGCAGACAAGTCTGGTCGAGCTGG	AAATCCAGCTCGACCAGACTTGTCTG
**On-target T25**	CGTGCAGACAAGTTCGACCAGGATGG	AAATCCATCCTGGTCGAACTTGTCTG
**On-target T26**	CGTGCAGACAAGTTCAGCGTGTCCGG	AAATCCGGACACGCTGAACTTGTCTG
**On-target T27**	CGTGCAGACAAGTAGGGTGGGCCAGG	AAATCCTGGCCCACCCTACTTGTCTG
**On-target T28**	CGTGCAGACAAGTGTCACGAGGGTGG	AAATCCACCCTCGTGACACTTGTCTG
**On-target T29**	CGTGCAGACAAGTGTGGTCACGAGGG	AAATCCCTCGTGACCACACTTGTCTG
**On-target T30**	CGTGCAGACAAGTCCGTAGGTCAGGG	AAATCCCTGACCTACGGACTTGTCTG
**On-target T31**	CGTGCAGACAAGTGTCGGGGTAGCGG	AAATCCGCTACCCCGACACTTGTCTG
**On-target T32**	CGTGCAGACAAGTATCGAGCTGAAGG	AAATCCTTCAGCTCGATACTTGTCTG
**On-target T33**	CGTGCAGACAAGTGACTTCAAGGAGG	AAATCCTCCTTGAAGTCACTTGTCTG
**On-target T34**	CGTGCAGACAAGTGTCGCCGATGGGG	AAATCCCCATCGGCGACACTTGTCTG
**Oligonucleotides used for cloning on-target screen sgRNA plasmids**		
**plasmid name**	**oligo1**	**oligo2**
**sgRNA - On-target T1**	CACCGCAGACAAGTGCATAGACTG	AAACCAGTCTATGCACTTGTCTGC
**sgRNA - On-target T2**	CACCGCAGACAAGTAGCTTGCCGG	AAACCCGGCAAGCTACTTGTCTGC
**sgRNA - On-target T3**	CACCGCAGACAAGTGCGACGTAAA	AAACTTTACGTCGCACTTGTCTGC
**sgRNA - On-target T4**	CACCGCAGACAAGTATGAACTTCA	AAACTGAAGTTCATACTTGTCTGC
**sgRNA - On-target T5**	CACCGCAGACAAGTATCTTCTTCA	AAACTGAAGAAGATACTTGTCTGC

**sgRNA - On-target T6**	CACCGCAGACAAGTAACTTCACCT	AAACAGGTGAAGTTACTTGTCTGC
**sgRNA - On-target T7**	CACCGCAGACAAGTATGGTCCTGC	AAACGCAGGACCATACTTGTCTGC
**sgRNA - On-target T8**	CACCGCAGACAAGTAGAGTGATCC	AAACGGATCACTCTACTTGTCTGC
**sgRNA - On-target T9**	CACCGCAGACAAGTTTGAAGAAGA	AAACTCTTCTTCAAACTTGTCTGC
**sgRNA - On-target T10**	CACCGCAGACAAGTGGCGACACCC	AAACGGGTGTCGCCACTTGTCTGC
**sgRNA - On-target T11**	CACCGCAGACAAGTAGGGCGGACT	AAACAGTCCGCCCTACTTGTCTGC
**sgRNA - On-target T12**	CACCGCAGACAAGTAGTGGTTGTC	AAACGACAACCACTACTTGTCTGC
**sgRNA - On-target T13**	CACCGCAGACAAGTTCGCCCTCGC	AAACGCGAGGGCGAACTTGTCTGC
**sgRNA - On-target T14**	CACCGCAGACAAGTATGCCACCTA	AAACTAGGTGGCATACTTGTCTGC
**sgRNA - On-target T15**	CACCGCAGACAAGTTGCACGCCGT	AAACACGGCGTGCAACTTGTCTGC
**sgRNA - On-target T16**	CACCGCAGACAAGTTCGAGCTGAA	AAACTTCAGCTCGAACTTGTCTGC
**sgRNA - On-target T17**	CACCGCAGACAAGTTCACGAGGGT	AAACACCCTCGTGAACTTGTCTGC
**sgRNA - On-target T18**	CACCGCAGACAAGTTCGGGCATGG	AAACCCATGCCCGAACTTGTCTGC
**sgRNA - On-target T19**	CACCGCAGACAAGTTCAAGGAGGA	AAACTCCTCCTTGAACTTGTCTGC
**sgRNA - On-target T20**	CACCGCAGACAAGTTGGTAGTGGT	AAACACCACTACCAACTTGTCTGC
**sgRNA - On-target T21**	CACCGCAGACAAGTGGGTGGGCCA	AAACTGGCCCACCCACTTGTCTGC
**sgRNA - On-target T22**	CACCGCAGACAAGTCTGTTCACCG	AAACCGGTGAACAGACTTGTCTGC
**sgRNA - On-target T23**	CACCGCAGACAAGTGGCACCACCC	AAACGGGTGGTGCCACTTGTCTGC
**sgRNA - On-target T24**	CACCGCAGACAAGTCTGGTCGAGC	AAACGCTCGACCAGACTTGTCTGC
**sgRNA - On-target T25**	CACCGCAGACAAGTTCGACCAGGA	AAACTCCTGGTCGAACTTGTCTGC
**sgRNA - On-target T26**	CACCGCAGACAAGTTCAGCGTGTC	AAACGACACGCTGAACTTGTCTGC
**sgRNA - On-target T27**	CACCGCAGACAAGTAGGGTGGGCC	AAACGGCCCACCCTACTTGTCTGC
**sgRNA - On-target T28**	CACCGCAGACAAGTGTCACGAGGG	AAACCCCTCGTGACACTTGTCTGC
**sgRNA - On-target T29**	CACCGCAGACAAGTGTGGTCACGA	AAACTCGTGACCACACTTGTCTGC
**sgRNA - On-target T30**	CACCGCAGACAAGTCCGTAGGTCA	AAACTGACCTACGGACTTGTCTGC
**sgRNA - On-target T31**	CACCGCAGACAAGTGTCGGGGTAG	AAACCTACCCCGACACTTGTCTGC
**sgRNA - On-target T32**	CACCGCAGACAAGTATCGAGCTGA	AAACTCAGCTCGATACTTGTCTGC
**sgRNA - On-target T33**	CACCGCAGACAAGTGACTTCAAGG	AAACCCTTGAAGTCACTTGTCTGC
**sgRNA - On-target T34**	CACCGCAGACAAGTGTCGCCGATG	AAACCATCGGCGACACTTGTCTGC
**Oligonucleotides used for cloning mismatching sgRNAs (Fig.6, Supplementary Fig.7)**		
**Target 1 mismatching sgRNAs (Fig. 6)**		
**plasmid name**	**oligo1**	**oligo2**
**T1-1MM1**	CACCACAGACAAGTGCATAGACTG	AAACCAGTCTATGCACTTGTCTGT

**T1-1MM2**	CACCGTAGACAAGTGCATAGACTG	AAACCAGTCTATGCACTTGTCTAC
**T1-1MM3**	CACCGCGGACAAGTGCATAGACTG	AAACCAGTCTATGCACTTGTCCGC
**T1-1MM4**	CACCGCAAACAAGTGCATAGACTG	AAACCAGTCTATGCACTTGTTTGC
**T1-1MM5**	CACCGCAGGCAAGTGCATAGACTG	AAACCAGTCTATGCACTTGCCTGC
**T1-1MM6**	CACCGCAGATAAGTGCATAGACTG	AAACCAGTCTATGCACTTATCTGC
**T1-1MM7**	CACCGCAGACGAGTGCATAGACTG	AAACCAGTCTATGCACTCGTCTGC
**T1-1MM8**	CACCGCAGACAGGTGCATAGACTG	AAACCAGTCTATGCACCTGTCTGC
**T1-1MM9**	CACCGCAGACAAATGCATAGACTG	AAACCAGTCTATGCATTTGTCTGC
**T1-1MM10**	CACCGCAGACAAGAGCATAGACTG	AAACCAGTCTATGCTCTTGTCTGC
**T1-1MM11**	CACCGCAGACAAGTACATAGACTG	AAACCAGTCTATGTACTTGTCTGC
**T1-1MM12**	CACCGCAGACAAGTGTATAGACTG	AAACCAGTCTATACACTTGTCTGC
**T1-1MM13**	CACCGCAGACAAGTGCGTAGACTG	AAACCAGTCTACGCACTTGTCTGC
**T1-1MM14**	CACCGCAGACAAGTGCAAAGACTG	AAACCAGTCTTTGCACTTGTCTGC
**T1-1MM15**	CACCGCAGACAAGTGCATGGACTG	AAACCAGTCCATGCACTTGTCTGC
**T1-1MM16**	CACCGCAGACAAGTGCATAAACTG	AAACCAGTTTATGCACTTGTCTGC
**T1-1MM17**	CACCGCAGACAAGTGCATAGGCTG	AAACCAGCCTATGCACTTGTCTGC
**T1-1MM18**	CACCGCAGACAAGTGCATAGATTG	AAACCAATCTATGCACTTGTCTGC
**T1-1MM19**	CACCGCAGACAAGTGCATAGACAG	AAACCTGTCTATGCACTTGTCTGC
**T1-1MM20**	CACCGCAGACAAGTGCATAGACTA	AAACTAGTCTATGCACTTGTCTGC
**T1-2MM1**	CACCATAGACAAGTGCATAGACTG	AAACCAGTCTATGCACTTGTCTAT
**T1-2MM2**	CACCGTGGACAAGTGCATAGACTG	AAACCAGTCTATGCACTTGTCCAC
**T1-2MM3**	CACCGCGAACAAGTGCATAGACTG	AAACCAGTCTATGCACTTGTTCGC
**T1-2MM5**	CACCGCAGGTAAGTGCATAGACTG	AAACCAGTCTATGCACTTACCTGC
**T1-2MM7**	CACCGCAGACGGGTGCATAGACTG	AAACCAGTCTATGCACCCGTCTGC
**T1-2MM9**	CACCGCAGACAAAAGCATAGACTG	AAACCAGTCTATGCTTTTGTCTGC
**T1-2MM11**	CACCGCAGACAAGTATATAGACTG	AAACCAGTCTATATACTTGTCTGC
**T1-2MM13**	CACCGCAGACAAGTGCGAAGACTG	AAACCAGTCTTCGCACTTGTCTGC
**T1-2MM15**	CACCGCAGACAAGTGCATGAACTG	AAACCAGTTCATGCACTTGTCTGC
**T1-2MM17**	CACCGCAGACAAGTGCATAGGTTG	AAACCAACCTATGCACTTGTCTGC
**T1-2MM19**	CACCGCAGACAAGTGCATAGACAA	AAACTTGTCTATGCACTTGTCTGC
**T1-3MM1**	CACCATGGACAAGTGCATAGACTG	AAACCAGTCTATGCACTTGTCCAT
**T1-3MM2**	CACCGTGAACAAGTGCATAGACTG	AAACCAGTCTATGCACTTGTTCAC
**T1-3MM3**	CACCGCGAGCAAGTGCATAGACTG	AAACCAGTCTATGCACTTGCTCGC
**T1-3MM6**	CACCGCAGATGGGTGCATAGACTG	AAACCAGTCTATGCACCCATCTGC
**T1-3MM9**	CACCGCAGACAAAAACATAGACTG	AAACCAGTCTATGTTTTTGTCTGC
**T1-3MM12**	CACCGCAGACAAGTGTGAAGACTG	AAACCAGTCTTCACACTTGTCTGC
**T1-3MM15**	CACCGCAGACAAGTGCATGAGCTG	AAACCAGCTCATGCACTTGTCTGC

**T1-3MM18**	CACCGCAGACAAGTGCATAGATAA	AAACTTATCTATGCACTTGTCTGC
**T1-4MM1**	CACCATGAACAAGTGCATAGACTG	AAACCAGTCTATGCACTTGTTCAT
**T1-4MM2**	CACCGTGAGCAAGTGCATAGACTG	AAACCAGTCTATGCACTTGCTCAC
**T1-4MM5**	CACCGCAGGTGGGTGCATAGACTG	AAACCAGTCTATGCACCCACCTGC
**T1-4MM9**	CACCGCAGACAAAAATATAGACTG	AAACCAGTCTATATTTTTGTCTGC
**T1-4MM13**	CACCGCAGACAAGTGCGAGAACTG	AAACCAGTTCTCGCACTTGTCTGC
**T1-4MM17**	CACCGCAGACAAGTGCATAGGTAA	AAACTTACCTATGCACTTGTCTGC
**T1-5MM1**	CACCATGAGCAAGTGCATAGACTG	AAACCAGTCTATGCACTTGCTCAT
**T1-5MM2**	CACCGTGAGTAAGTGCATAGACTG	AAACCAGTCTATGCACTTACTCAC
**T1-5MM6**	CACCGCAGATGGAAGCATAGACTG	AAACCAGTCTATGCTTCCATCTGC
**T1-5MM11**	CACCGCAGACAAGTATGAGGACTG	AAACCAGTCCTCATACTTGTCTGC
**T1-5MM16**	CACCGCAGACAAGTGCATAAGTAA	AAACTTACTTATGCACTTGTCTGC
**Target 2 mismatching sgRNAs (Supplementary Fig.7)**		
**plasmid name**	**oligo1**	**oligo2**
**T2-1MM1**	CACCACAGACAAGTAGCTTGCCGG	AAACCCGGCAAGCTACTTGTCTGT
**T2-1MM2**	CACCGTAGACAAGTAGCTTGCCGG	AAACCCGGCAAGCTACTTGTCTAC
**T2-1MM3**	CACCGCGGACAAGTAGCTTGCCGG	AAACCCGGCAAGCTACTTGTCCGC
**T2-1MM4**	CACCGCAAACAAGTAGCTTGCCGG	AAACCCGGCAAGCTACTTGTTTGC
**T2-1MM5**	CACCGCAGGCAAGTAGCTTGCCGG	AAACCCGGCAAGCTACTTGCCTGC
**T2-1MM6**	CACCGCAGATAAGTAGCTTGCCGG	AAACCCGGCAAGCTACTTATCTGC
**T2-1MM7**	CACCGCAGACGAGTAGCTTGCCGG	AAACCCGGCAAGCTACTCGTCTGC
**T2-1MM8**	CACCGCAGACAGGTAGCTTGCCGG	AAACCCGGCAAGCTACCTGTCTGC
**T2-1MM9**	CACCGCAGACAAATAGCTTGCCGG	AAACCCGGCAAGCTATTTGTCTGC
**T2-1MM10**	CACCGCAGACAAGCAGCTTGCCGG	AAACCCGGCAAGCTGCTTGTCTGC
**T2-1MM11**	CACCGCAGACAAGTGGCTTGCCGG	AAACCCGGCAAGCCACTTGTCTGC
**T2-1MM12**	CACCGCAGACAAGTAACTTGCCGG	AAACCCGGCAAGTTACTTGTCTGC
**T2-1MM13**	CACCGCAGACAAGTAGTTTGCCGG	AAACCCGGCAAACTACTTGTCTGC
**T2-1MM14**	CACCGCAGACAAGTAGCCTGCCGG	AAACCCGGCAGGCTACTTGTCTGC
**T2-1MM15**	CACCGCAGACAAGTAGCTCGCCGG	AAACCCGGCGAGCTACTTGTCTGC
**T2-1MM16**	CACCGCAGACAAGTAGCTTACCGG	AAACCCGGTAAGCTACTTGTCTGC
**T2-1MM17**	CACCGCAGACAAGTAGCTTGTCGG	AAACCCGACAAGCTACTTGTCTGC
**T2-1MM18**	CACCGCAGACAAGTAGCTTGCTGG	AAACCCAGCAAGCTACTTGTCTGC
**T2-1MM19**	CACCGCAGACAAGTAGCTTGCCAG	AAACCTGGCAAGCTACTTGTCTGC

**T2-1MM20**	CACCGCAGACAAGTAGCTTGCCGA	AAACTCGGCAAGCTACTTGTCTGC
**T2-2MM1**	CACCATAGACAAGTAGCTTGCCGG	AAACCCGGCAAGCTACTTGTCTAT
**T2-2MM2**	CACCGTGGACAAGTAGCTTGCCGG	AAACCCGGCAAGCTACTTGTCCAC
**T2-2MM3**	CACCGCGAACAAGTAGCTTGCCGG	AAACCCGGCAAGCTACTTGTTCGC
**T2-2MM5**	CACCGCAGGTAAGTAGCTTGCCGG	AAACCCGGCAAGCTACTTACCTGC
**T2-2MM7**	CACCGCAGACGGGTAGCTTGCCGG	AAACCCGGCAAGCTACCCGTCTGC
**T2-2MM9**	CACCGCAGACAAACAGCTTGCCGG	AAACCCGGCAAGCTGTTTGTCTGC
**T2-2MM11**	CACCGCAGACAAGTGACTTGCCGG	AAACCCGGCAAGTCACTTGTCTGC
**T2-2MM13**	CACCGCAGACAAGTAGTCTGCCGG	AAACCCGGCAGACTACTTGTCTGC
**T2-2MM15**	CACCGCAGACAAGTAGCTCACCGG	AAACCCGGTGAGCTACTTGTCTGC
**T2-2MM17**	CACCGCAGACAAGTAGCTTGTTGG	AAACCCAACAAGCTACTTGTCTGC
**T2-2MM19**	CACCGCAGACAAGTAGCTTGCCAA	AAACTTGGCAAGCTACTTGTCTGC
**Target 6 mismatching sgRNAs (Supplementary Fig.7)**		
**plasmid name**	**oligo1**	**oligo2**
**T6-1MM1**	CACCACAGACAAGTAACTTCACCT	AAACAGGTGAAGTTACTTGTCTGT
**T6-1MM2**	CACCGTAGACAAGTAACTTCACCT	AAACAGGTGAAGTTACTTGTCTAC
**T6-1MM3**	CACCGCGGACAAGTAACTTCACCT	AAACAGGTGAAGTTACTTGTCCGC
**T6-1MM4**	CACCGCAAACAAGTAACTTCACCT	AAACAGGTGAAGTTACTTGTTTGC
**T6-1MM5**	CACCGCAGGCAAGTAACTTCACCT	AAACAGGTGAAGTTACTTGCCTGC
**T6-1MM6**	CACCGCAGATAAGTAACTTCACCT	AAACAGGTGAAGTTACTTATCTGC
**T6-1MM7**	CACCGCAGACGAGTAACTTCACCT	AAACAGGTGAAGTTACTCGTCTGC
**T6-1MM8**	CACCGCAGACAGGTAACTTCACCT	AAACAGGTGAAGTTACCTGTCTGC
**T6-1MM9**	CACCGCAGACAAATAACTTCACCT	AAACAGGTGAAGTTATTTGTCTGC
**T6-1MM10**	CACCGCAGACAAGCAACTTCACCT	AAACAGGTGAAGTTGCTTGTCTGC
**T6-1MM11**	CACCGCAGACAAGTGACTTCACCT	AAACAGGTGAAGTCACTTGTCTGC
**T6-1MM12**	CACCGCAGACAAGTAGCTTCACCT	AAACAGGTGAAGCTACTTGTCTGC
**T6-1MM13**	CACCGCAGACAAGTAATTTCACCT	AAACAGGTGAAATTACTTGTCTGC
**T6-1MM14**	CACCGCAGACAAGTAACCTCACCT	AAACAGGTGAGGTTACTTGTCTGC
**T6-1MM15**	CACCGCAGACAAGTAACTCCACCT	AAACAGGTGGAGTTACTTGTCTGC
**T6-1MM16**	CACCGCAGACAAGTAACTTTACCT	AAACAGGTAAAGTTACTTGTCTGC
**T6-1MM17**	CACCGCAGACAAGTAACTTCGCCT	AAACAGGCGAAGTTACTTGTCTGC
**T6-1MM18**	CACCGCAGACAAGTAACTTCATCT	AAACAGATGAAGTTACTTGTCTGC
**T6-1MM19**	CACCGCAGACAAGTAACTTCACTT	AAACAAGTGAAGTTACTTGTCTGC
**T6-1MM20**	CACCGCAGACAAGTAACTTCACCC	AAACGGGTGAAGTTACTTGTCTGC

**T6-2MM1**	CACCATAGACAAGTAACTTCACCT	AAACAGGTGAAGTTACTTGTCTAT
**T6-2MM2**	CACCGTGGACAAGTAACTTCACCT	AAACAGGTGAAGTTACTTGTCCAC
**T6-2MM3**	CACCGCGAACAAGTAACTTCACCT	AAACAGGTGAAGTTACTTGTTCGC
**T6-2MM5**	CACCGCAGGTAAGTAACTTCACCT	AAACAGGTGAAGTTACTTACCTGC
**T6-2MM7**	CACCGCAGACGGGTAACTTCACCT	AAACAGGTGAAGTTACCCGTCTGC
**T6-2MM9**	CACCGCAGACAAACAACTTCACCT	AAACAGGTGAAGTTGTTTGTCTGC
**T6-2MM11**	CACCGCAGACAAGTGGCTTCACCT	AAACAGGTGAAGCCACTTGTCTGC
**T6-2MM13**	CACCGCAGACAAGTAATCTCACCT	AAACAGGTGAGATTACTTGTCTGC
**T6-2MM15**	CACCGCAGACAAGTAACTCTACCT	AAACAGGTAGAGTTACTTGTCTGC
**T6-2MM17**	CACCGCAGACAAGTAACTTCGTCT	AAACAGACGAAGTTACTTGTCTGC
**T6-2MM19**	CACCGCAGACAAGTAACTTCACTC	AAACGAGTGAAGTTACTTGTCTGC
**Target 17 mismatching sgRNAs (Supplementary Fig.7)**		
**plasmid name**	**oligo1**	**oligo2**
**T17-1MM1**	CACCACAGACAAGTTCACGAGGGT	AAACACCCTCGTGAACTTGTCTGT
**T17-1MM2**	CACCGTAGACAAGTTCACGAGGGT	AAACACCCTCGTGAACTTGTCTAC
**T17-1MM3**	CACCGCGGACAAGTTCACGAGGGT	AAACACCCTCGTGAACTTGTCCGC
**T17-1MM4**	CACCGCAAACAAGTTCACGAGGGT	AAACACCCTCGTGAACTTGTTTGC
**T17-1MM5**	CACCGCAGGCAAGTTCACGAGGGT	AAACACCCTCGTGAACTTGCCTGC
**T17-1MM6**	CACCGCAGATAAGTTCACGAGGGT	AAACACCCTCGTGAACTTATCTGC
**T17-1MM7**	CACCGCAGACGAGTTCACGAGGGT	AAACACCCTCGTGAACTCGTCTGC
**T17-1MM8**	CACCGCAGACAGGTTCACGAGGGT	AAACACCCTCGTGAACCTGTCTGC
**T17-1MM9**	CACCGCAGACAAATTCACGAGGGT	AAACACCCTCGTGAATTTGTCTGC
**T17-1MM10**	CACCGCAGACAAGCTCACGAGGGT	AAACACCCTCGTGAGCTTGTCTGC
**T17-1MM11**	CACCGCAGACAAGTCCACGAGGGT	AAACACCCTCGTGGACTTGTCTGC
**T17-1MM12**	CACCGCAGACAAGTTTACGAGGGT	AAACACCCTCGTAAACTTGTCTGC
**T17-1MM13**	CACCGCAGACAAGTTCGCGAGGGT	AAACACCCTCGCGAACTTGTCTGC
**T17-1MM14**	CACCGCAGACAAGTTCATGAGGGT	AAACACCCTCATGAACTTGTCTGC
**T17-1MM15**	CACCGCAGACAAGTTCACAAGGGT	AAACACCCTTGTGAACTTGTCTGC
**T17-1MM16**	CACCGCAGACAAGTTCACGGGGGT	AAACACCCCCGTGAACTTGTCTGC
**T17-1MM17**	CACCGCAGACAAGTTCACGAAGGT	AAACACCTTCGTGAACTTGTCTGC
**T17-1MM18**	CACCGCAGACAAGTTCACGAGAGT	AAACACTCTCGTGAACTTGTCTGC
**T17-1MM19**	CACCGCAGACAAGTTCACGAGGAT	AAACATCCTCGTGAACTTGTCTGC
**T17-1MM20**	CACCGCAGACAAGTTCACGAGGGC	AAACGCCCTCGTGAACTTGTCTGC
**T17-2MM1**	CACCATAGACAAGTTCACGAGGGT	AAACACCCTCGTGAACTTGTCTAT

**T17-2MM2**	CACCGTGGACAAGTTCACGAGGGT	AAACACCCTCGTGAACTTGTCCAC
**T17-2MM3**	CACCGCGAACAAGTTCACGAGGGT	AAACACCCTCGTGAACTTGTTCGC
**T17-2MM5**	CACCGCAGGTAAGTTCACGAGGGT	AAACACCCTCGTGAACTTACCTGC
**T17-2MM7**	CACCGCAGACGGGTTCACGAGGGT	AAACACCCTCGTGAACCCGTCTGC
**T17-2MM9**	CACCGCAGACAAACTCACGAGGGT	AAACACCCTCGTGAGTTTGTCTGC
**T17-2MM11**	CACCGCAGACAAGTCTACGAGGGT	AAACACCCTCGTAGACTTGTCTGC
**T17-2MM13**	CACCGCAGACAAGTTCGTGAGGGT	AAACACCCTCACGAACTTGTCTGC
**T17-2MM15**	CACCGCAGACAAGTTCACAGGGGT	AAACACCCCTGTGAACTTGTCTGC
**T17-2MM17**	CACCGCAGACAAGTTCACGAAAGT	AAACACTTTCGTGAACTTGTCTGC
**T17-2MM19**	CACCGCAGACAAGTTCACGAGGAC	AAACGTCCTCGTGAACTTGTCTGC
**Oligos for cloning BEAR-GFP-2in1**		
BEAR-GFP-2in1-for	gcgcacatcgcccacagtcCGTTACATAACTTACGGTAAATG	
BEAR-GFP-2in1-rev	TTGCTGGCCTTTTGCTCAgcTAAGATACATTGATGAGTTTGGAC	

Supplementary Table 3 – List of NGS primers and NGS indexing

**Genomic primers for NGS (1st step PCR)**		
**Amplicon**	**Fwd (i5) primer**	**Rev (i7) primer**
HEK-SITE1	TCGTCGGCAGCGTCAGATGTGTATAAGAGACAGTCCCCTGTAGTTTCGGCAATAG	GTCTCGTGGGCTCGGAGATGTGTATAAGAGACAGCTGGAGGGCAAGTGCTGTCT
HEK-SITE2	TCGTCGGCAGCGTCAGATGTGTATAAGAGACAGGTCCAGCCCCATCTGTCAAA	GTCTCGTGGGCTCGGAGATGTGTATAAGAGACAGAGGACGTCTGCCCAATATGT
HEK-SITE3	TCGTCGGCAGCGTCAGATGTGTATAAGAGACAGCCCAGCCAAACTTGTCAACC	GTCTCGTGGGCTCGGAGATGTGTATAAGAGACAGGGGAAACGCCCATGCAATTA
HEK-SITE4	TCGTCGGCAGCGTCAGATGTGTATAAGAGACAGTCCTTTCAACCCGAACGGAG	GTCTCGTGGGCTCGGAGATGTGTATAAGAGACAGGGAACCCAGGTAGCCAGAGAC
CCR5	TCGTCGGCAGCGTCAGATGTGTATAAGAGACAGTTCCTGGGAGAGACGCAAAC	GTCTCGTGGGCTCGGAGATGTGTATAAGAGACAGTTCTGGGCTCACTATGCTGC
FANCF-SITE2	TCGTCGGCAGCGTCAGATGTGTATAAGAGACAGGGTGCTGACGTAGGTAGTGC	GTCTCGTGGGCTCGGAGATGTGTATAAGAGACAGACACGGATAAAGACGCTGGG
SCN5a	TCGTCGGCAGCGTCAGATGTGTATAAGAGACAGGGCAAACTTCCTATTACCTCGGG	GTCTCGTGGGCTCGGAGATGTGTATAAGAGACAGCCCAGAGCCTCATGAGCCAC

**Indexing primers (2nd step PCR)**	
i5 indexing primer	AATGATACGGCGACCACCGAGATCTACAC-*i5 index*-TCGTCGGCAGCGTC
i7 indexing primer	CAAGCAGAAGACGGCATACGAGAT-*i7 index*-GTCTCGTGGGCTCGG

**i5 and i7 indices**	
i5-S513	TCGACTAG
i5-S515	TTCTAGCT
i5-S516	CCTAGAGT
i5-S518	CTATTAAG
i5-S520	AAGGCTAT
i5-S521	GAGCCTTA
i5-S522	TTATGCGA
i7-N701	TCGCCTTA
i7-N702	CTAGTACG
i7-N703	TTCTGCCT
i7-N704	GCTCAGGA
i7-N705	AGGAGTCC
i7-N706	CATGCCTA
i7-N707	GTAGAGAG
i7-N710	CAGCCTCG
i7-N711	TGCCTCTT
i7-N712	TCCTCTAC
i7-N714	TCATGAGC
i7-N715	CCTGAGAT

Sample indexing for NGS						
**Sample name**	**Base editor**	**Enrichment**	**Parallel experiment**	**Amplicon**	**i5 index**	**i7 index**
1	ABE	No enrichment	1	HEK-SITE1	S513	N701
2	ABE	Transfection enrichment	1	HEK-SITE1	S513	N702
3	ABE	BEAR enrichment	1	HEK-SITE1	S513	N703
4	ABE	No enrichment	2	HEK-SITE1	S513	N704
5	ABE	Transfection enrichment	2	HEK-SITE1	S513	N705
6	ABE	BEAR enrichment	2	HEK-SITE1	S513	N706
7	ABE	No enrichment	3	HEK-SITE1	S513	N707
8	ABE	Transfection enrichment	3	HEK-SITE1	S513	N710
9	ABE	BEAR enrichment	3	HEK-SITE1	S513	N711
10	dABE	No enrichment	1	HEK-SITE1	S513	N712
11	dABE	Transfection enrichment	1	HEK-SITE1	S513	N714
12	dABE	BEAR enrichment	1	HEK-SITE1	S513	N715
13	dABE	No enrichment	2	HEK-SITE1	S515	N701
14	dABE	Transfection enrichment	2	HEK-SITE1	S515	N702
15	dABE	BEAR enrichment	2	HEK-SITE1	S515	N703
16	dABE	No enrichment	3	HEK-SITE1	S515	N704
17	dABE	Transfection enrichment	3	HEK-SITE1	S515	N705
18	dABE	BEAR enrichment	3	HEK-SITE1	S515	N706
19	no base editor	N/A	1	HEK-SITE1	S515	N707
20	no base editor	N/A	2	HEK-SITE1	S515	N710
21	no base editor	N/A	3	HEK-SITE1	S515	N711
22	ABE	No enrichment	1	HEK-SITE2	S515	N712
23	ABE	Transfection enrichment	1	HEK-SITE2	S515	N714
24	ABE	BEAR enrichment	1	HEK-SITE2	S515	N715
25	ABE	No enrichment	2	HEK-SITE2	S516	N701
26	ABE	Transfection enrichment	2	HEK-SITE2	S516	N702
27	ABE	BEAR enrichment	2	HEK-SITE2	S516	N703
28	ABE	No enrichment	3	HEK-SITE2	S516	N704
29	ABE	Transfection enrichment	3	HEK-SITE2	S516	N705
30	ABE	BEAR enrichment	3	HEK-SITE2	S516	N706
31	dABE	No enrichment	1	HEK-SITE2	S516	N707
32	dABE	Transfection enrichment	1	HEK-SITE2	S516	N710
33	dABE	BEAR enrichment	1	HEK-SITE2	S516	N711
34	dABE	No enrichment	2	HEK-SITE2	S516	N712
35	dABE	Transfection enrichment	2	HEK-SITE2	S516	N714
36	dABE	BEAR enrichment	2	HEK-SITE2	S516	N715
37	dABE	No enrichment	3	HEK-SITE2	S518	N701
38	dABE	Transfection enrichment	3	HEK-SITE2	S518	N702
39	dABE	BEAR enrichment	3	HEK-SITE2	S518	N703
40	no base editor	N/A	1	HEK-SITE2	S518	N704
41	no base editor	N/A	2	HEK-SITE2	S518	N705
42	no base editor	N/A	3	HEK-SITE2	S518	N706
43	ABE	No enrichment	1	HEK-SITE3	S518	N707
44	ABE	Transfection enrichment	1	HEK-SITE3	S518	N710
45	ABE	BEAR enrichment	1	HEK-SITE3	S518	N711
46	ABE	No enrichment	2	HEK-SITE3	S518	N712
47	ABE	Transfection enrichment	2	HEK-SITE3	S518	N714
48	ABE	BEAR enrichment	2	HEK-SITE3	S518	N715
49	ABE	No enrichment	3	HEK-SITE3	S520	N701
50	ABE	Transfection enrichment	3	HEK-SITE3	S520	N702

51	ABE	BEAR enrichment	3	HEK-SITE3	S520	N703
52	dABE	No enrichment	1	HEK-SITE3	S520	N704
53	dABE	Transfection enrichment	1	HEK-SITE3	S520	N705
54	dABE	BEAR enrichment	1	HEK-SITE3	S520	N706
55	dABE	No enrichment	2	HEK-SITE3	S520	N707
56	dABE	Transfection enrichment	2	HEK-SITE3	S520	N710
57	dABE	BEAR enrichment	2	HEK-SITE3	S520	N711
58	dABE	No enrichment	3	HEK-SITE3	S520	N712
59	dABE	Transfection enrichment	3	HEK-SITE3	S520	N714
60	dABE	BEAR enrichment	3	HEK-SITE3	S520	N715
61	CBE	No enrichment	1	HEK-SITE3	S521	N701
62	CBE	No enrichment	2	HEK-SITE3	S521	N702
63	CBE	No enrichment	3	HEK-SITE3	S521	N703
64	dCBE	No enrichment	1	HEK-SITE3	S521	N704
65	dCBE	Transfection enrichment	1	HEK-SITE3	S521	N705
66	dCBE	BEAR enrichment	1	HEK-SITE3	S521	N706
67	dCBE	No enrichment	2	HEK-SITE3	S521	N707
68	dCBE	Transfection enrichment	2	HEK-SITE3	S521	N710
69	dCBE	BEAR enrichment	2	HEK-SITE3	S521	N711
70	dCBE	No enrichment	3	HEK-SITE3	S521	N712
71	dCBE	Transfection enrichment	3	HEK-SITE3	S521	N714
72	dCBE	BEAR enrichment	3	HEK-SITE3	S521	N715
73	no base editor	N/A	1	HEK-SITE3	S522	N701
74	no base editor	N/A	2	HEK-SITE3	S522	N702
75	no base editor	N/A	3	HEK-SITE3	S522	N703
76	CBE	No enrichment	1	HEK-SITE4	S522	N704
77	CBE	No enrichment	2	HEK-SITE4	S522	N705
78	CBE	No enrichment	3	HEK-SITE4	S522	N706
79	dCBE	No enrichment	1	HEK-SITE4	S522	N707
80	dCBE	Transfection enrichment	1	HEK-SITE4	S522	N710
81	dCBE	BEAR enrichment	1	HEK-SITE4	S522	N711
82	dCBE	No enrichment	2	HEK-SITE4	S522	N712
83	dCBE	Transfection enrichment	2	HEK-SITE4	S522	N714
84	dCBE	BEAR enrichment	2	HEK-SITE4	S513	N701
85	dCBE	No enrichment	3	HEK-SITE4	S513	N702
86	dCBE	Transfection enrichment	3	HEK-SITE4	S513	N703
87	dCBE	BEAR enrichment	3	HEK-SITE4	S513	N704
88	no base editor	N/A	1	HEK-SITE4	S513	N705
89	no base editor	N/A	2	HEK-SITE4	S513	N706
90	no base editor	N/A	3	HEK-SITE4	S513	N707
91	ABE	No enrichment	1	CCR5	S513	N710
92	ABE	Transfection enrichment	1	CCR5	S513	N711
93	ABE	BEAR enrichment	1	CCR5	S513	N712
94	ABE	No enrichment	2	CCR5	S513	N714
95	ABE	Transfection enrichment	2	CCR5	S513	N715
96	ABE	BEAR enrichment	2	CCR5	S515	N701
97	ABE	No enrichment	3	CCR5	S515	N702
98	ABE	Transfection enrichment	3	CCR5	S515	N703
99	ABE	BEAR enrichment	3	CCR5	S515	N704
100	dABE	No enrichment	1	CCR5	S515	N705
101	dABE	Transfection enrichment	1	CCR5	S515	N706
102	dABE	BEAR enrichment	1	CCR5	S515	N707
103	dABE	No enrichment	2	CCR5	S515	N710
104	dABE	Transfection enrichment	2	CCR5	S515	N711
105	dABE	BEAR enrichment	2	CCR5	S515	N712
106	dABE	No enrichment	3	CCR5	S515	N714
107	dABE	Transfection enrichment	3	CCR5	S515	N715
108	dABE	BEAR enrichment	3	CCR5	S516	N701
109	CBE	No enrichment	1	CCR5	S516	N702

110	CBE	No enrichment	2	CCR5	S516	N703
111	CBE	No enrichment	3	CCR5	S516	N704
112	dCBE	No enrichment	1	CCR5	S516	N705
113	dCBE	Transfection enrichment	1	CCR5	S516	N706
114	dCBE	BEAR enrichment	1	CCR5	S516	N707
115	dCBE	No enrichment	2	CCR5	S516	N710
116	dCBE	Transfection enrichment	2	CCR5	S516	N711
117	dCBE	BEAR enrichment	2	CCR5	S516	N712
118	dCBE	No enrichment	3	CCR5	S516	N714
119	dCBE	Transfection enrichment	3	CCR5	S516	N715
120	dCBE	BEAR enrichment	3	CCR5	S518	N701
121	no base editor	N/A	1	CCR5	S518	N702
122	no base editor	N/A	2	CCR5	S518	N703
123	no base editor	N/A	3	CCR5	S518	N704
124	CBE	No enrichment	1	FANCF-SITE2	S518	N705
125	CBE	No enrichment	2	FANCF-SITE2	S518	N706
126	CBE	No enrichment	3	FANCF-SITE2	S518	N707
127	dCBE	No enrichment	1	FANCF-SITE2	S518	N710
128	dCBE	Transfection enrichment	1	FANCF-SITE2	S518	N711
129	dCBE	BEAR enrichment	1	FANCF-SITE2	S518	N712
130	dCBE	No enrichment	2	FANCF-SITE2	S518	N714
131	dCBE	Transfection enrichment	2	FANCF-SITE2	S518	N715
132	dCBE	BEAR enrichment	2	FANCF-SITE2	S520	N701
133	dCBE	No enrichment	3	FANCF-SITE2	S520	N702
134	dCBE	Transfection enrichment	3	FANCF-SITE2	S520	N703
135	dCBE	BEAR enrichment	3	FANCF-SITE2	S520	N704
136	no base editor	N/A	1	FANCF-SITE2	S520	N705
137	no base editor	N/A	2	FANCF-SITE2	S520	N706
138	no base editor	N/A	3	FANCF-SITE2	S520	N707
139	ABE	No enrichment	1	SCN5a	S520	N710
140	ABE	Transfection enrichment	1	SCN5a	S520	N711
141	ABE	BEAR enrichment	1	SCN5a	S520	N712
142	ABE	No enrichment	2	SCN5a	S520	N714
143	ABE	Transfection enrichment	2	SCN5a	S520	N715
144	ABE	BEAR enrichment	2	SCN5a	S521	N701
145	ABE	No enrichment	3	SCN5a	S521	N702
146	ABE	Transfection enrichment	3	SCN5a	S521	N703
147	ABE	BEAR enrichment	3	SCN5a	S521	N704
148	dABE	No enrichment	1	SCN5a	S521	N705
149	dABE	Transfection enrichment	1	SCN5a	S521	N706
150	dABE	BEAR enrichment	1	SCN5a	S521	N707
151	dABE	No enrichment	2	SCN5a	S521	N710
152	dABE	Transfection enrichment	2	SCN5a	S521	N711
153	dABE	BEAR enrichment	2	SCN5a	S521	N712
154	dABE	No enrichment	3	SCN5a	S521	N714
155	dABE	Transfection enrichment	3	SCN5a	S521	N715
156	dABE	BEAR enrichment	3	SCN5a	S522	N701
157	CBE	No enrichment	1	SCN5a	S522	N702
158	CBE	No enrichment	2	SCN5a	S522	N703
159	CBE	No enrichment	3	SCN5a	S522	N704
160	dCBE	No enrichment	1	SCN5a	S522	N705
161	dCBE	Transfection enrichment	1	SCN5a	S522	N706
162	dCBE	BEAR enrichment	1	SCN5a	S522	N707
163	dCBE	No enrichment	2	SCN5a	S522	N710
164	dCBE	Transfection enrichment	2	SCN5a	S522	N711
165	dCBE	BEAR enrichment	2	SCN5a	S522	N712
166	dCBE	No enrichment	3	SCN5a	S522	N714
167	dCBE	Transfection enrichment	3	SCN5a	S522	N715
168	dCBE	BEAR enrichment	3	SCN5a	S513	N701

169	no base editor	N/A	1	SCN5a	S513	N702
170	no base editor	N/A	2	SCN5a	S513	N703
171	no base editor	N/A	3	SCN5a	S513	N704
