# Supplementary material for: BEAR reveals that increased fidelity variants can successfully reduce the mismatch tolerance of adenine but not cytosine base editors
Source: Nat Commun. 2021 Nov 3;12:6353. doi: 10.1038/s41467-021-26461-y (PMC8566456; doi:10.1038/s41467-021-26461-y)
Supplement: Supplementary file 3 — Reporting Summary [file 41467_2021_26461_MOESM3_ESM.pdf]

nature portfolio

Corresponding author(s): András Tálas, Ervin Welker

Last updated by author(s): Sep 20, 2021

Reporting Summary

Nature Portfolio wishes to improve the reproducibility of the work that we publish. This form provides structure for consistency and transparency in reporting. For further information on Nature Portfolio policies, see our Editorial Policies and the Editorial Policy Checklist.

Statistics

For all statistical analyses, confirm that the following items are present in the figure legend, table legend, main text, or Methods section.

n/a	Confirmed	
☐	☒	The exact sample size (n) for each experimental group/condition, given as a discrete number and unit of measurement
☐	☒	A statement on whether measurements were taken from distinct samples or whether the same sample was measured repeatedly
☐	☒	The statistical test(s) used AND whether they are one- or two-sided<br>*Only common tests should be described solely by name; describe more complex techniques in the Methods section.*
☒	☐	A description of all covariates tested
☐	☒	A description of any assumptions or corrections, such as tests of normality and adjustment for multiple comparisons
☐	☒	A full description of the statistical parameters including central tendency (e.g. means) or other basic estimates (e.g. regression coefficient) AND variation (e.g. standard deviation) or associated estimates of uncertainty (e.g. confidence intervals)
☐	☒	For null hypothesis testing, the test statistic (e.g. F, t, r) with confidence intervals, effect sizes, degrees of freedom and P value noted<br>*Give P values as exact values whenever suitable.*
☒	☐	For Bayesian analysis, information on the choice of priors and Markov chain Monte Carlo settings
☒	☐	For hierarchical and complex designs, identification of the appropriate level for tests and full reporting of outcomes
☐	☒	Estimates of effect sizes (e.g. Cohen's d, Pearson's r), indicating how they were calculated

Our web collection on statistics for biologists contains articles on many of the points above.

Software and code

Policy information about availability of computer code

Data collection	Flow cytometry data collection was carried out on Attune NxT Acoustic Focusing Cytometer (Applied Biosystems) using Attune NxT Software v.4.2. Cell sorting was performed on FACSAriaIII (BD Biosciences) using BD FACSDiva Software. NGS data was collected on Illumina NextSeq instrument.
Data analysis	Targeted deep-sequencing data were analysed using a the following softwares: BBMap 38.08, samtools 1.8, BioPython 1.71, PySam 0.13. Editing efficiencies (on Fig. 3c,d) were analysed by EditR 1.0.9 (https://moriaritylab.shinyapps.io/editr_v10/). Indels were counted computationally among the aligned reads that matched at least 75% to the first 20 bp of the reference amplicon. Indels without mismatches were searched at ±2bp around the cut site with allowing indels of any size. For each sample, indel frequency was determined as (number of reads with an indel)/(number of total reads). For each sample, base editing frequency was determined as the percentage of all sequencing reads with a target adenine converted to guanine (in the case of ABEs) or as the percentage of sequencing reads with a target cytosine converted to thymine (in the case of CBEs). To avoid falsely high specificity ratios on Figure 4e and 4h, during calculations indels lower than 0.05% were assumed to be 0.05% as this amount is considered to be the resolution limit of NGS. Statistical analyses were performed using IBM SPSS (ver. 20). For FACS data analysis Attune NxT Software v.4.2 was used.

For manuscripts utilizing custom algorithms or software that are central to the research but not yet described in published literature, software must be made available to editors and reviewers. We strongly encourage code deposition in a community repository (e.g. GitHub). See the Nature Portfolio guidelines for submitting code & software for further information.

Data

Policy information about availability of data

All manuscripts must include a data availability statement. This statement should provide the following information, where applicable:

- Accession codes, unique identifiers, or web links for publicly available datasets
- A description of any restrictions on data availability
- For clinical datasets or third party data, please ensure that the statement adheres to our policy

All NGS data is available in the NCBI Sequence Read Archive under accession number PRJNA748771. Relevant plasmids are available from Addgene (detailed in the plasmid construction section).

Field-specific reporting

Please select the one below that is the best fit for your research. If you are not sure, read the appropriate sections before making your selection.

☒ Life sciences ☐ Behavioural & social sciences ☐ Ecological, evolutionary & environmental sciences

For a reference copy of the document with all sections, see nature.com/documents/nr-reporting-summary-flat.pdf

Life sciences study design

All studies must disclose on these points even when the disclosure is negative.

Sample size	No statistical methods were used to predetermine or justify sample size, but each condition was performed in tripliate which is generally accepted sample size for similar gene editing experiments.
Data exclusions	No data were excluded.
Replication	Independent replicates (n=3) were performed. All attempts at replication were successful.
Randomization	Not relevant to these experiments.
Blinding	Not relevant to these experiments.

Reporting for specific materials, systems and methods

We require information from authors about some types of materials, experimental systems and methods used in many studies. Here, indicate whether each material, system or method listed is relevant to your study. If you are not sure if a list item applies to your research, read the appropriate section before selecting a response.

Materials & experimental systems

n/a	Involved in the study	
☒	☐	Antibodies
☐	☒	Eukaryotic cell lines
☒	☐	Palaeontology and archaeology
☒	☐	Animals and other organisms
☒	☐	Human research participants
☒	☐	Clinical data
☒	☐	Dual use research of concern

Methods

n/a	Involved in the study	
☒	☐	ChIP-seq
☐	☒	Flow cytometry
☒	☐	MRI-based neuroimaging

Eukaryotic cell lines

Policy information about cell lines

Cell line source(s)	N2a (neuro-2a mouse neuroblastoma cells, CCL-131) and HEK293T (CRL-1573) were from ATCC, BEAR-mScarlet and BEAR-GFP cell lines were cloned by us.
Authentication	Cell lines were not authenticated as they were obtained directly from a certified repository or clone from those cell lines.
Mycoplasma contamination	Cells were tested monthly for mycoplasma contamination with negative test results.
Commonly misidentified lines (See ICLAC register)	No commonly misidentified cell lines were used.

Flow Cytometry

Plots

Confirm that:

- [x] The axis labels state the marker and fluorochrome used (e.g. CD4-FITC).
- [x] The axis scales are clearly visible. Include numbers along axes only for bottom left plot of group (a 'group' is an analysis of identical markers).
- [x] All plots are contour plots with outliers or pseudocolor plots.
- [x] A numerical value for number of cells or percentage (with statistics) is provided.

Methodology

Sample preparation	Cells were washed with PBS and after that trypsinized for 1 min. Completed DMEM was added after cells were separated (confirmed by microscope).
Instrument	Flow cytometry analysis was carried out using an Attune NxT Acoustic Focusing Cytometer (Applied Biosystems by Life Technologies). Cell sorting was carried out on a FACSAria III cell sorter (BD Biosciences).
Software	Attune Cytometric Software v.4.2, BD FACSDiva Software
Cell population abundance	In flow cytometry experiments a minimum of 10,000 live single cells were aquired. In cell sorting experiments a minimum of 50,000 cells were sorted in all experiments.
Gating strategy	Negative controls (cells not transfected) were used to establish GFP, mScarlet, BFP and mCherry +/- gates. In cell sorting experiments to sort control (no enrichment) cells, live single cells were sorted regardless of any fluorescent markers. To sort transfection marker enriched cells, BFP positive cells were sorted regardless of GFP fluorescence. To sort BEAR enriched cells GFP positive cells, were sorted regardless of BFP fluorescence. Detalied gating strategy is shown on Supplementary Figure 11.

- [x] Tick this box to confirm that a figure exemplifying the gating strategy is provided in the Supplementary Information.
